# Supplementary material for: CFP1 governs uterine epigenetic landscapes to intervene in progesterone responses for uterine physiology and suppression of endometriosis
Source: Nat Commun. 2023 Jun 3;14:3220. doi: 10.1038/s41467-023-39008-0 (PMC10239508; doi:10.1038/s41467-023-39008-0)
Supplement: Supplementary file 1 — Supplementary information [file 41467_2023_39008_MOESM1_ESM.pdf]

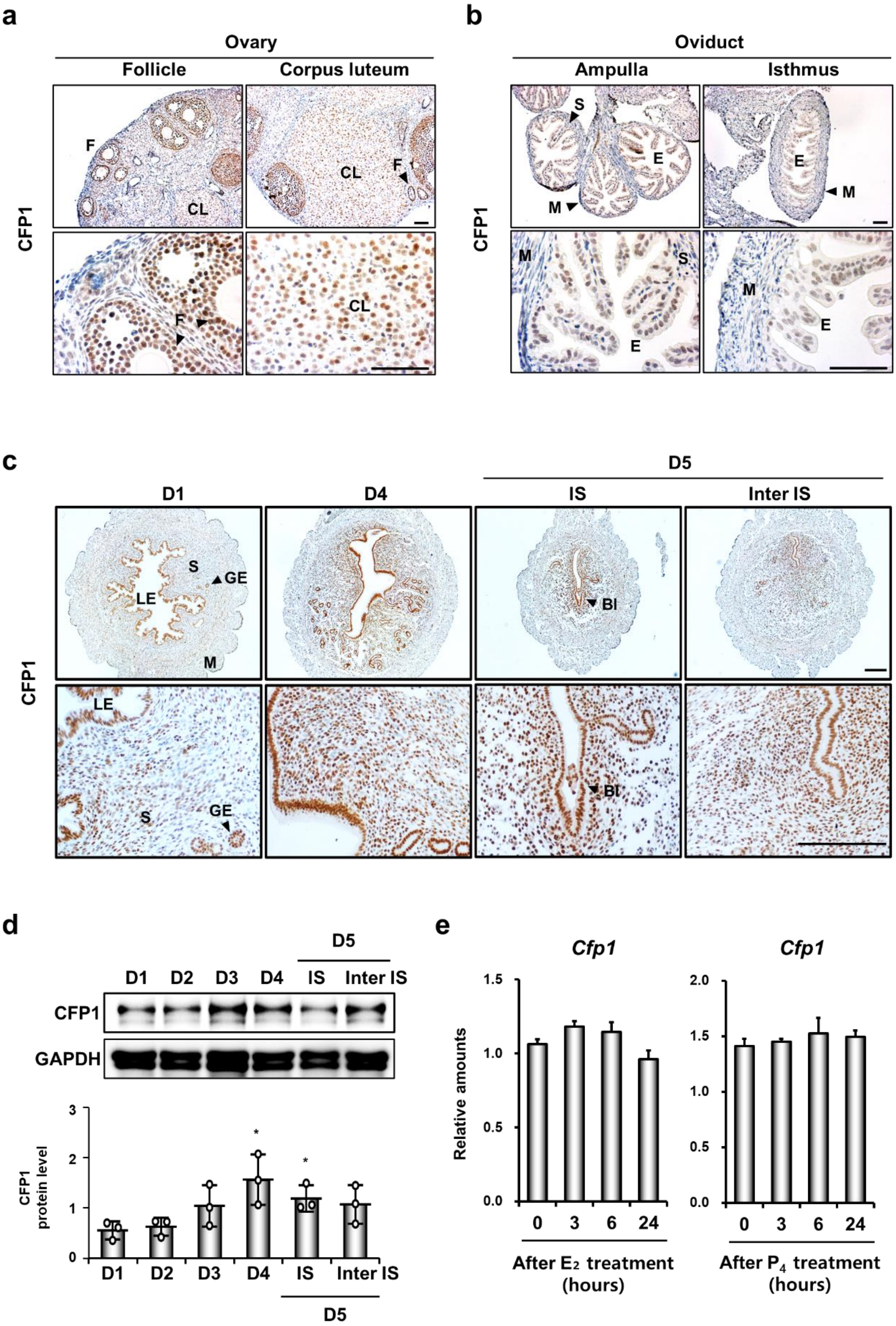

a
Ovary
Follicle
Corpus luteum
CFP1
F
CL
b
Oviduct
Ampulla
Isthmus
S
M
E
c
D1
D4
D5
IS
Inter IS
LE
GE
Bl
d
CFP1
GAPDH
CFP1 protein level
D1
D2
D3
D4
e
Cfp1
Relative amounts
After E2 treatment (hours)
After P4 treatment (hours)
0
3
6
24

Supplementary Fig 1. Immunohistochemical localization of CFP1 protein in the reproductive tracts of female mice. Immunohistochemical staining for CFP1 in the ovary (a), oviduct (b), and uterus (c). The bottom panel presents high-power images of the top panel. Scale bars, 200 μm. CL, corpus luteum; F, follicle; M, muscle cells; S, stroma; E, epithelium; GE, glandular epithelium; LE, luminal epithelium. (d) Western blotting for CFP1 protein expression in mouse uterus during early pregnancy. n=3 biologically independent samples per group. Data are presented as mean values with SD. Statistical analyses were performed using the multiple comparisons. *p < 0.05. (e) Real-time RT-PCR analyses for relative mRNA levels of *Cfp1* after E_2 or P_4 treatment in the uterus of OVX mice at a different time point. n=4-5 biologically independent samples per group. Data are presented as mean values with SD.

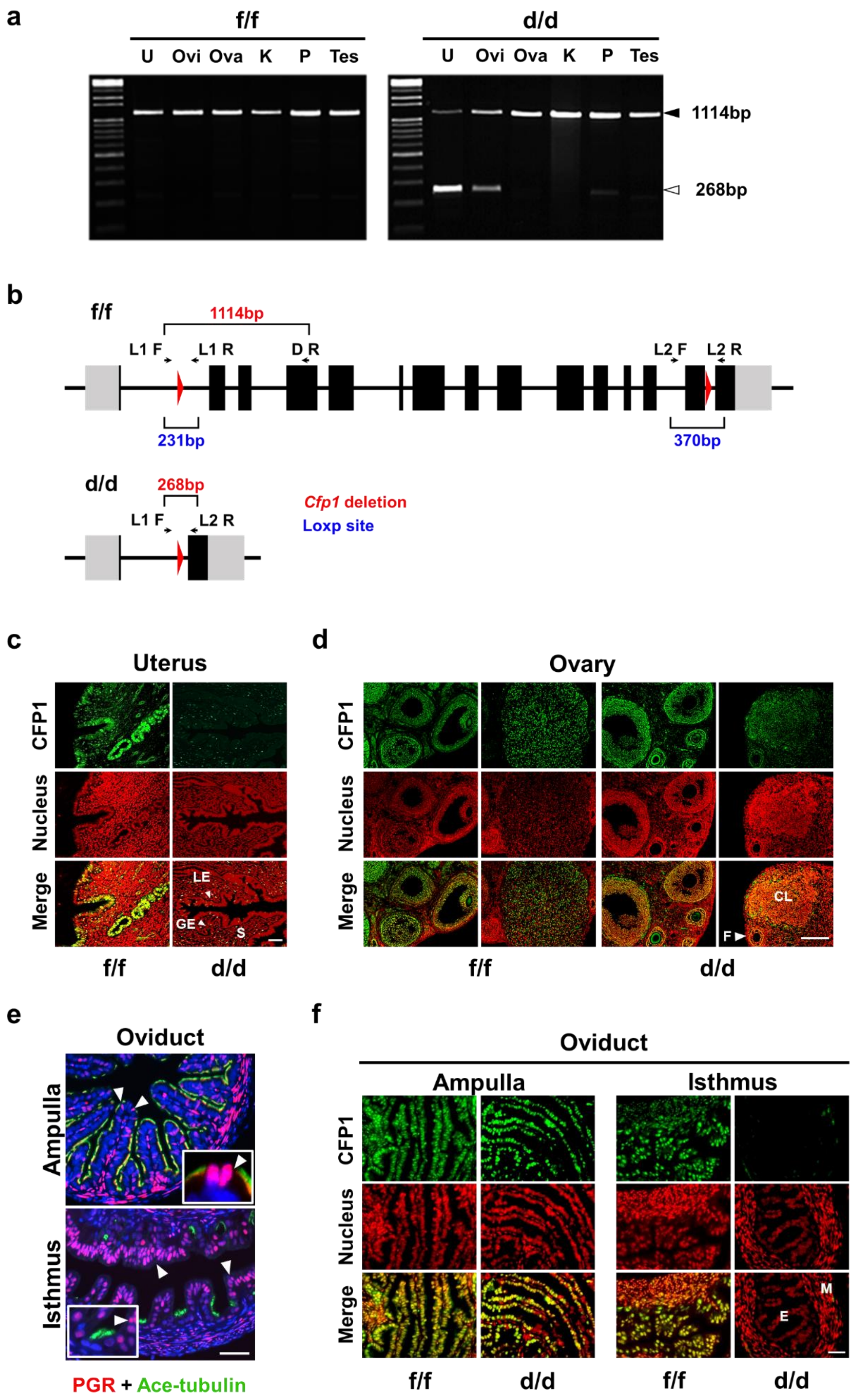
a
f/f
d/d
U Ovi Ova K P Tes
U Ovi Ova K P Tes
1114bp
268bp
b
f/f
1114bp
L1 F L1 R D R L2 F L2 R
231bp
370bp
d/d
268bp
L1 F L2 R
Cfp1 deletion
Loxp site
c
Uterus
CFP1
Nucleus
Merge
LE
GE
S
f/f d/d
d
Ovary
CFP1
Nucleus
Merge
CL
F
f/f d/d
e
Oviduct
Ampulla
Isthmus
PGR + Ace-tubulin
f
Oviduct
Ampulla
Isthmus
CFP1
Nucleus
Merge
M
E
f/f d/d f/f d/d

Supplementary Fig 2. Genetic deletion of the *Cfp1* gene in female reproductive tracts of *Cfp1*$^{d/d}$ mice. (a) Representative images of PCR results with the genomic DNA of various tissues. Black and white arrowheads indicate PCR products for inclusion (1114 bp) and deletion (268 bp) of *Cfp1* exons 2–14, respectively. K, kidney; Ova, ovary; Ovi, oviduct; P, pituitary; Tes, testis; U, uterus. (b) Schematic of PCR primers used to detect the structures of the conditional *Cfp1* allele (*Cfp1*$^{f/f}$) and disrupted *Cfp1* allele (*Cfp1*$^{d/d}$). (c-d) Immunofluorescence staining for CFP1 in the uterus (c) and ovary (d) of *Cfp1*$^{f/f}$ and *Cfp1*$^{d/d}$ mice. (e) Immunofluorescence staining for acetylated tubulin (cilia marker, white arrowhead) and PGR in the oviduct of *Cfp1*$^{d/d}$ mouse on Day 4. Scale bar, 50 μm (f) Immunofluorescence staining for CFP1 in the oviduct of *Cfp1*$^{f/f}$ and *Cfp1*$^{d/d}$ mice. CL, corpus luteum; F, follicle; E, epithelium; GE, glandular epithelium; LE, luminal epithelium; S, stroma; M, muscle. Scale bar: uterus, 100 μm; ovary, 200 μm; oviduct, 200 μm.

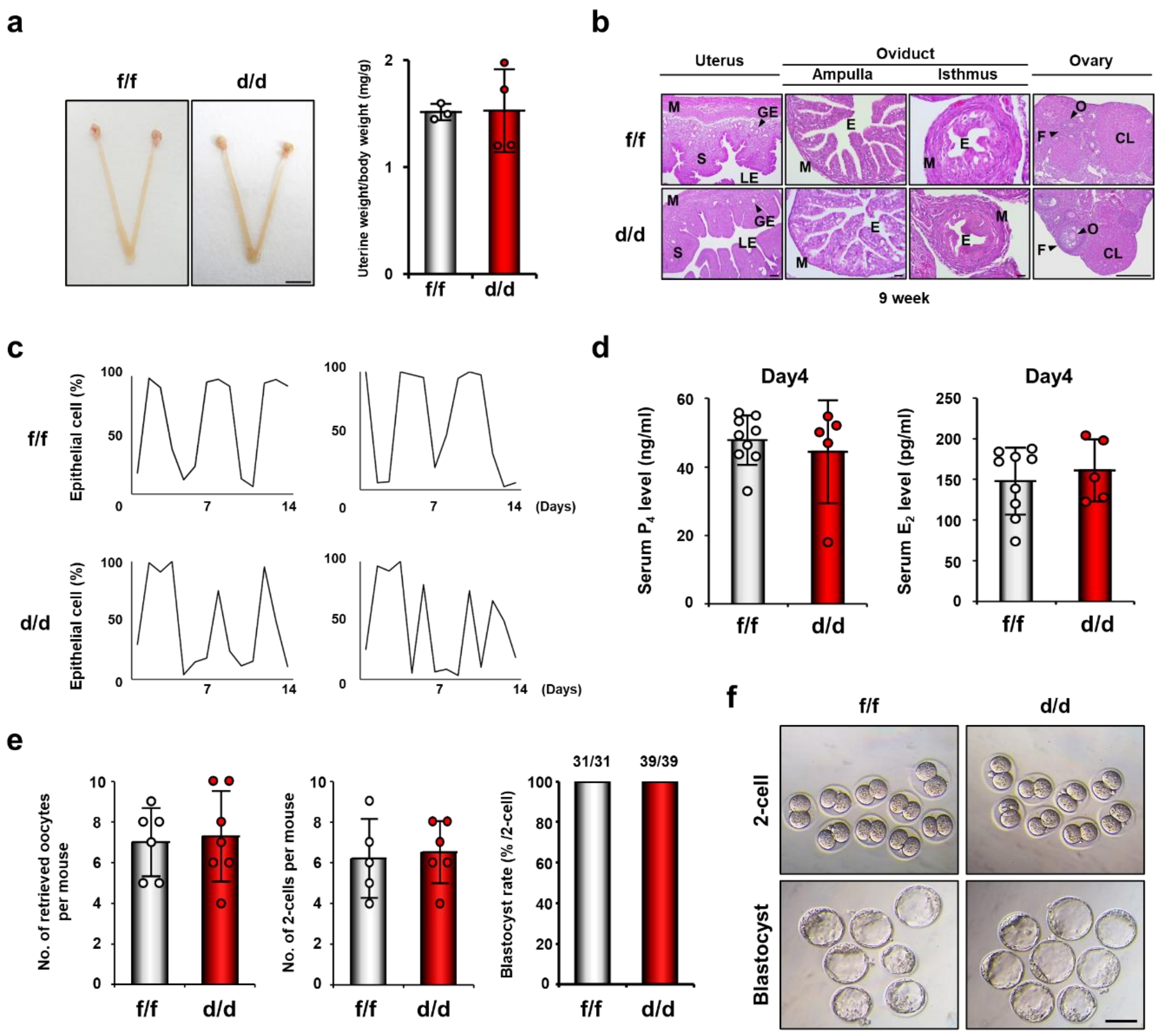
a
f/f
d/d
Uterine weight/body weight (mg/g)
2
1
0
f/f
d/d
b
Uterus
Oviduct
Ampulla
Isthmus
Ovary
f/f
d/d
M
GE
S
LE
E
F
O
CL
9 week
c
f/f
d/d
Epithelial cell (%)
100
50
0
7
14
(Days)
d
Day4
Serum P4 level (ng/ml)
Serum E2 level (pg/ml)
60
40
20
0
250
200
150
100
50
f/f
d/d
e
No. of retrieved oocytes per mouse
No. of 2-cells per mouse
Blastocyst rate (% /2-cell)
10
8
6
4
2
0
31/31
39/39
100
80
60
40
20
f/f
d/d
f
f/f
d/d
2-cell
Blastocyst

Supplementary Fig 3. Gross architectures and function of the female reproductive tracts of *Cfp1*$^{d/d}$ mice. (a) Gross morphology of the female reproductive tracts and uterine weight/total body weight in 8-week-old *Cfp1*$^{f/f}$ and *Cfp1*$^{d/d}$ mice. Scale bar, 5 mm. (b) Hematoxylin and eosin staining of the uterus, oviduct, and ovary in *Cfp1*$^{f/f}$ and *Cfp1*$^{d/d}$ mice. n=4 biologically independent samples per genotype. Scale bars: uterus and ovary, 200 µm; oviduct, 50 µm. CL, corpus luteum; F, follicle; S, stroma; M, muscle cells; E, epithelium; GE, glandular epithelium; LE, luminal epithelium; O, oocyte. (c) Representative graphs to demonstrate % change of epithelial cell/total cells examined by a vaginal smear method for 2 weeks in *Cfp1*$^{f/f}$ and *Cfp1*$^{d/d}$ mice. (d) Measurement of E_2 and P_4 levels in the serum of *Cfp1*$^{f/f}$ and *Cfp1*$^{d/d}$ mice on Day 4. n=5 to 9 biologically independent samples per genotype. Data are presented as mean values with SD. (e) The ovulation, fertilization, and *in vitro* embryo development in *Cfp1*$^{d/d}$ mice. The graphs present the average number of ovulated oocytes (left) and fertilized 2-cell embryos (middle) per mouse in each genotype on Day 2, and *in vitro* blastocyst formation rate (%) of 2-cell embryos (right). n=5 to 7 biologically independent samples per genotype. Data are presented as mean values with SD. (f) Microscopic images of 2-cells and blastocysts developed *in vitro* from the 2-cells harvested from the oviducts of *Cfp1*$^{f/f}$ and *Cfp1*$^{d/d}$ mice on Day 2. Scale bar, 50 µm.

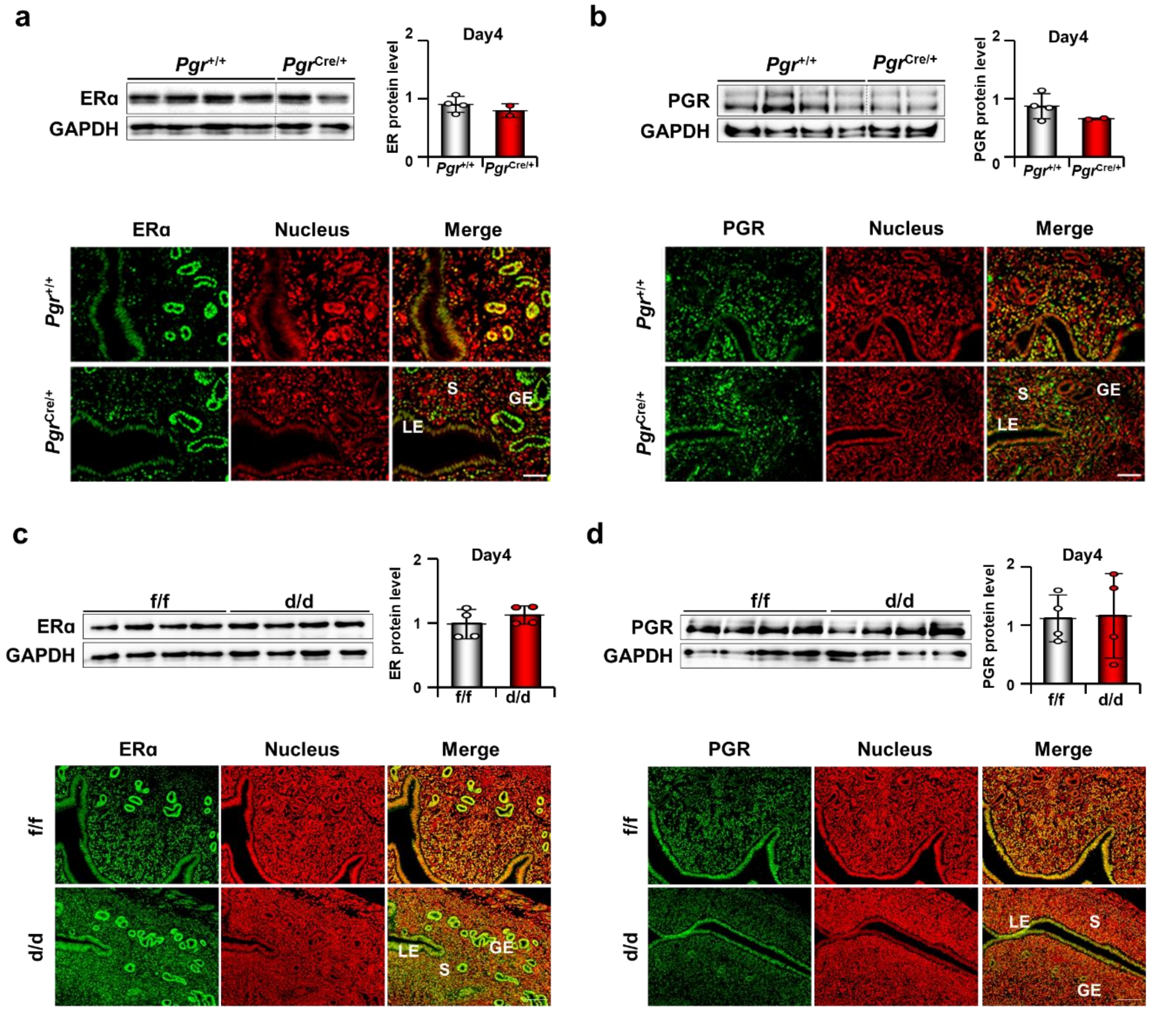
a
Pgr+/+
PgrCre/+
ERα
GAPDH
Day4
ER protein level
2
1
0
Pgr+/+
PgrCre/+
ERα
Nucleus
Merge
Pgr+/+
PgrCre/+
S
GE
LE
b
Pgr+/+
PgrCre/+
PGR
GAPDH
Day4
PGR protein level
2
1
0
Pgr+/+
PgrCre/+
PGR
Nucleus
Merge
Pgr+/+
PgrCre/+
S
GE
LE
c
f/f
d/d
ERα
GAPDH
Day4
ER protein level
2
1
0
f/f
d/d
ERα
Nucleus
Merge
f/f
d/d
LE
GE
S
d
f/f
d/d
PGR
GAPDH
Day4
PGR protein level
2
1
0
f/f
d/d
PGR
Nucleus
Merge
f/f
d/d
LE
S
GE

Supplementary Fig 4. Expression of ERα and PGR in *Pgr*$^{cre/+}$ and *Cfp1*$^{d/d}$ mouse uterus. (a-b) Western blotting and immunofluorescence staining for ERα (a) and PGR (b) in *Pgr*$^{+/+}$ and *Pgr*$^{Cre/+}$ mouse uterus on Day 4. n=2 to 4 biologically independent samples per genotype. Data are presented as mean values with SD. (c-d) Western blotting and immunofluorescence staining for ERα (c) and PGR (d) in *Cfp1*$^{f/f}$ and *Cfp1*$^{d/d}$ mouse uterus on Day 4. n=4 biologically independent samples per genotype. Data are presented as mean values with SD. GAPDH serves as a loading control. Scale bar, 100 μm. GE, glandular epithelium; LE, luminal epithelium; S, stroma.

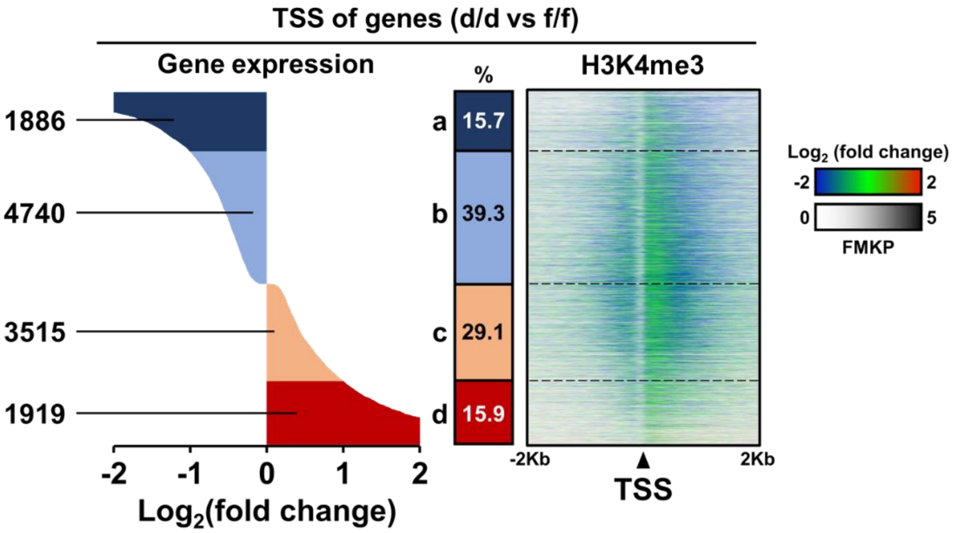

TSS of genes (d/d vs f/f)
Gene expression
%
H3K4me3
1886
4740
3515
1919
a
15.7
b
39.3
c
29.1
d
15.9
-2
-1
0
1
2
Log2(fold change)
-2Kb
2Kb
TSS
Log2 (fold change)
-2
2
0
5
FMKP

Supplementary Fig 5. Correlation of H3K4me3 levels with expression levels of DEGs in the uterus of *Cfp1*$^{d/d}$ mice on Day 4. All differentially regulated genes with statistical significance (left; adjusted $p < 0.05$), including heatmap of the associated H3K4me3 changes across such genes (Log_2 fold change $-1 > a$; $-1 < b < 0$; $0 < c < 1$; $1 < d$) in *Cfp1*$^{f/f}$ and *Cfp1*$^{d/d}$ uterus on Day 4 inherited H3K4me3 changes at such genes (right). The ChIP signal is expressed as shown in a ratio with strength in FPKM.

a

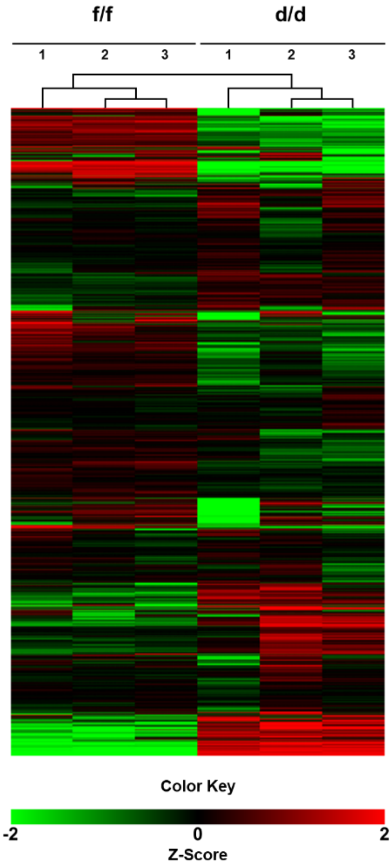

b

Upregulated GO

Rank	Name	Size	NES	FDR q-val
1	Keratin filament	32	2.26	0.00
2	Desmosome	23	2.11	0.01
3	Isoprenoid biosynthetic process	22	1.83	0.23
4	Defense response to virus	103	1.82	0.24
5	RHO guanyl nucleotide exchange factor activity	65	1.82	0.22
6	Cornified envelope	16	1.81	0.22

Downregulated GO

Rank	Name	Size	NES	FDR q-val
1	Regulation of smoothened signaling pathway	55	-2.06	0.03
2	Mesenchyme morphogenesis	37	-2.05	0.02
3	Adenylate cyclase inhibiting g protein coupled receptor signaling pathway	56	-2.05	0.01
4	Negative regulation of smoothened signaling pathway	24	-1.98	0.04
5	Spinal cord development	81	-1.98	0.03
6	Regulation of chondrocyte differentiation	41	-1.97	0.03
7	Regulation of cartilage development	56	-1.97	0.03
8	Regulation of systemic arterial blood pressure	72	-1.94	0.05
9	Adenylate cyclase modulating g protein coupled receptor signaling pathway	112	-1.93	0.04
10	Cytosolic large ribosomal subunit	54	-1.91	0.06
11	Response to platelet derived growth factor	18	-1.87	0.10
12	Ventricular septum development	49	-1.87	0.09
13	Synaptic signaling	365	-1.86	0.10
14	Retina vasculature development in camera type eye	16	-1.85	0.10
15	Regulation of dopamine secretion	20	-1.85	0.10
16	Cardiac septum development	77	-1.84	0.09
17	Nuclear transcribed mRNA catabolic process nonsense mediated decay	109	-1.84	0.09
18	Negative regulation of calcium ion transport	40	-1.83	0.10
19	Cytosolic ribosome	97	-1.83	0.09
20	Epithelial to mesenchymal transition	52	-1.82	0.11

Supplementary Fig 6. Unsupervised hierarchical clustering and the top 20 gene set list of mRNA-seq analysis in *Cfp1*$^{f/f}$ and *Cfp1*$^{d/d}$ mouse uterus on Day 4. (a) Unsupervised hierarchical clustering of RNA-seq analysis in *Cfp1*$^{f/f}$ and *Cfp1*$^{d/d}$ mouse uterus on Day 4. (b) Tables of the top 20 list of upregulated or downregulated GO term in Fig. 2f.

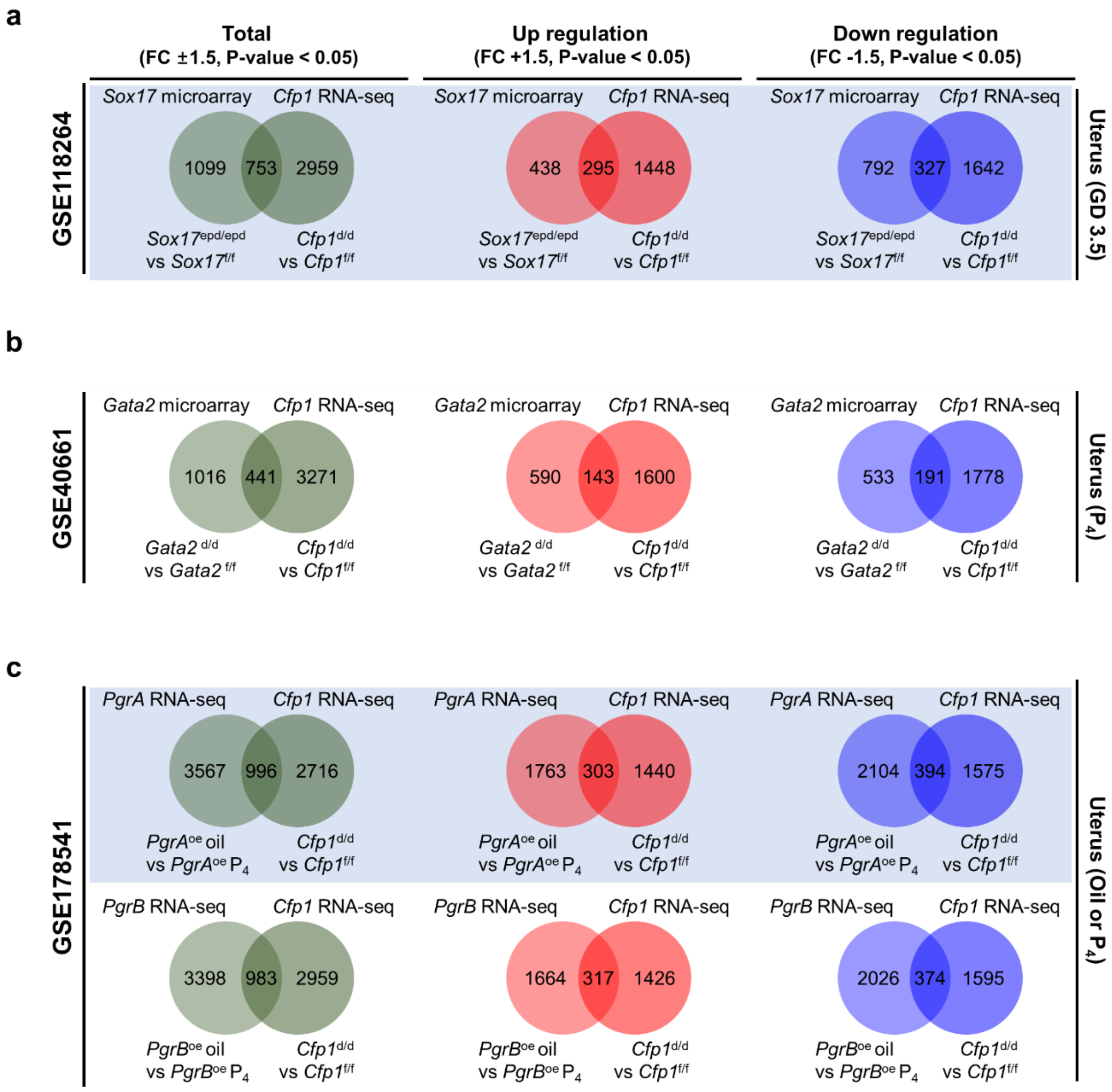
a
Total
(FC ±1.5, P-value < 0.05)
Up regulation
(FC +1.5, P-value < 0.05)
Down regulation
(FC -1.5, P-value < 0.05)
GSE118264
Uterus (GD 3.5)
Sox17 microarray
Cfp1 RNA-seq
1099
753
2959
438
295
1448
792
327
1642
Sox17 epd/epd vs Sox17 f/f
Cfp1 d/d vs Cfp1 f/f
b
GSE40661
Uterus (P4)
Gata2 microarray
Cfp1 RNA-seq
1016
441
3271
590
143
1600
533
191
1778
Gata2 d/d vs Gata2 f/f
Cfp1 d/d vs Cfp1 f/f
c
GSE178541
Uterus (Oil or P4)
PgrA RNA-seq
Cfp1 RNA-seq
3567
996
2716
1763
303
1440
2104
394
1575
PgrA oe oil vs PgrA oe P4
Cfp1 d/d vs Cfp1 f/f
PgrB RNA-seq
Cfp1 RNA-seq
3398
983
2959
1664
317
1426
2026
374
1595
PgrB oe oil vs PgrB oe P4
Cfp1 d/d vs Cfp1 f/f

Supplementary Fig 7. Venn diagrams of comparative analyses of *Cfp1*$^{d/d}$ mRNA-seq with other P_4 signaling-related transcriptomic datasets. (a-c) Intersections between DEGs of *Cfp1*$^{d/d}$ (*Pgr*-Cre) and *Sox17*$^{epd/epd}$ (*Ltf*-Cre) mouse uterus on Day 4 (GSE118264) (a), *Cfp1*$^{d/d}$ (*Pgr*-Cre) and P_4-treated *Gata2*$^{d/d}$ (*Pgr*-Cre) mouse uterus (GSE40661) (b), and *Cfp1*$^{d/d}$ (*Pgr*-Cre) and oil- or P_4-treated *PgrA*^{oe} (*Wnt7a*-Cre;*PgrA*$^{LsL/+}$) and *PgrB*^{oe} (*Wnt7a*-Cre;*PgrB*$^{LsL/+}$) mouse uterus (GSE178541) (c). epd, epithelial cell deletion; oe, overexpression.

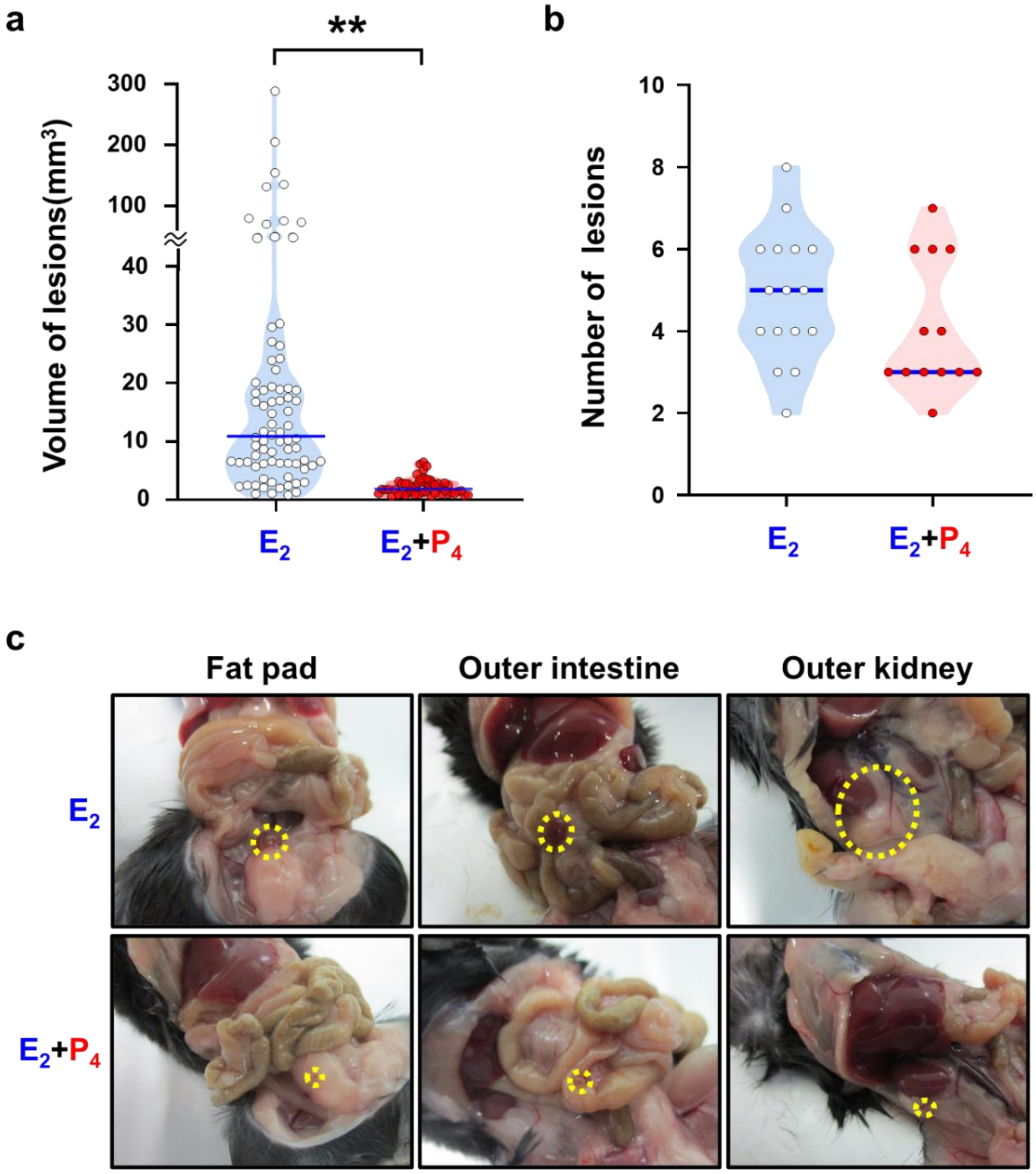
a
**
Volume of lesions(mm3)
300
200
100
40
30
20
10
0
E2
E2+P4
b
Number of lesions
10
8
6
4
2
0
E2
E2+P4
c
Fat pad
Outer intestine
Outer kidney
E2
E2+P4

Supplementary Fig 8. Establishment of a mouse model of endometriosis. (a-b) Average volumes (a) and numbers (b) of ectopic lesions found 15 days after transplantation were identified in recipients treated with E_2 or $E_2 + P_4$. n=13 to 16 biologically independent samples per group. Data are presented as mean values with SD. Statistical analyses were performed using the unpaired Student's t-tests. $^{**}p < 0.01$. (c) Representative images to show the location of ectopic lesions (dashed yellow circles).

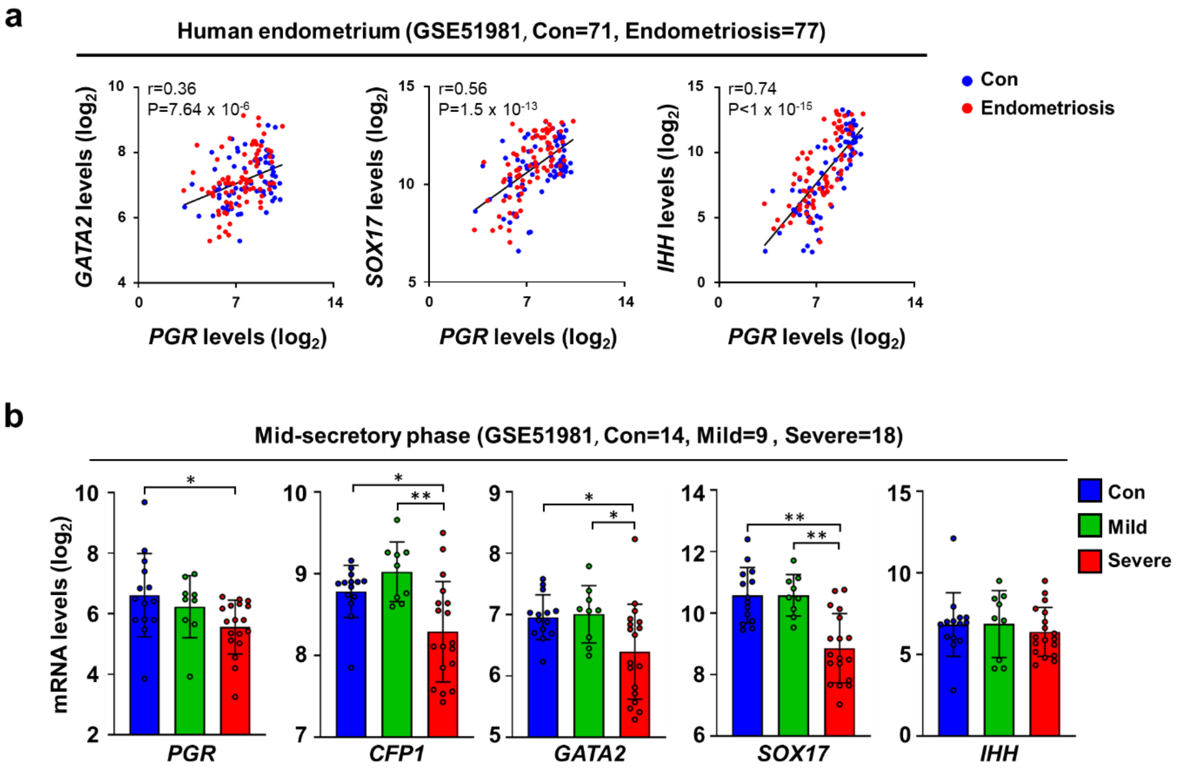
a
Human endometrium (GSE51981, Con=71, Endometriosis=77)
r=0.36
P=7.64 x 10-6
r=0.56
P=1.5 x 10-13
r=0.74
P<1 x 10-15
GATA2 levels (log2)
SOX17 levels (log2)
IHH levels (log2)
PGR levels (log2)
Con
Endometriosis
b
Mid-secretory phase (GSE51981, Con=14, Mild=9 , Severe=18)
mRNA levels (log2)
PGR
CFP1
GATA2
SOX17
IHH
Con
Mild
Severe

Supplementary Fig 9. Correlation of mRNA expression levels between *PGR* and key P_4-PGR downstream genes in SSP in human endometrium. (a) Correlations between expression levels of *PGR* and *GATA2*, *SOX17*, and *IHH* in human endometrium (data from GSE51981). (b) *PGR, CFP1, GATA2, SOX17,* and *IHH* mRNA expression levels (log_2) for control women and patients with mild or severe endometriosis from RNA-seq dataset (GSE51981). n=9 to 18 biologically independent samples per group. Data are presented as mean values with SD. Statistical analyses were performed using the multiple comparisons. *p < 0.05, **p < 0.01.

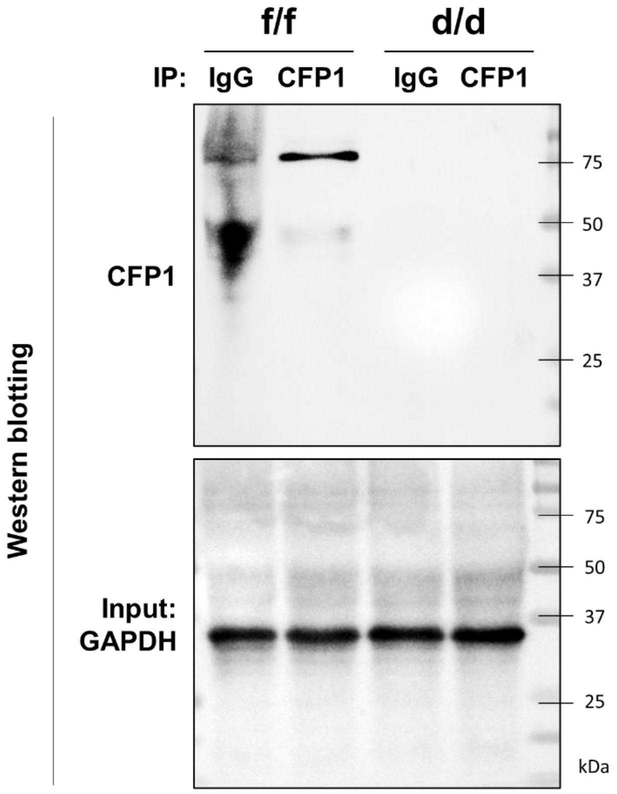
f/f
d/d
IP: IgG CFP1 IgG CFP1
Western blotting
CFP1
Input: GAPDH
75
50
37
25
75
50
37
25
kDa

Supplementary Fig 10. CFP1 immunoprecipitation and Western blotting in the uterus of *Cfp1*$^{f/f}$ and *Cfp1*$^{d/d}$ mice on Day 4. CFP1 Western blotting following immunoprecipitation with IgG or CFP1 antibodies for the uterus of *Cfp1*$^{f/f}$ or *Cfp1*$^{d/d}$ mice on Day 4. GAPDH serves as a loading control with input lysates.

Supplementary Table 1. Primer sequences for ChIP qPCR

Gene		Sequence (5'-3')	Position	Size (bp)
Gata2 CBS	Forward	ACCATTATTGGTCTAGCACAGCC	-574/-458	116
	Reverse	GTGCAAGAGGCTGGTAGGATC		
Sox17 CBS	Forward	GCGGATTAGGCGAAGGGTTAG	-112/-26	86
	Reverse	GGAGATGCCCAGAGGAACTCG		
Ihh CBS	Forward	TGAGATTACTTTACATGCTAGGC	-1665/-1561	104
	Reverse	CCTGGAATGATGTCTGCTAGG		
Ihh TSS	Forward	GGAATCCTTGAGCTTGGTAGCACGG	+1449/+1582	133
	Reverse	GATGTCTGGTGGAGTTGGGTCC		

CBS, CFP1 binding site; TSS, Transcription start site

Supplementary Table 2. Pregnancy outcomes of *Cfp1*$^{f/f}$; *LysM*$^{cre/+}$ female mice

Genotype	No. of mice	Litter size	Total No. of pups
Cfp1$^{f/f}$	2	9	18
Cfp1$^{f/f}$; *LysM*$^{Cre/+}$	3	11.3	34

Supplementary Table 3. Primer sequences for genotyping

Genotype		Sequence (5'-3')	Size (bp)
Cfp1$^{f/f}$	LoxP Forward 1	TGTAGACACACTTGTGGGAAGCC	WT: 231
	LoxP Reverse 1	ACTGTTTAGCCATCTCCC	f/f: 265
	LoxP Forward 2	AGTTCACCCAGACCCTCTTCC	WT: 370
	LoxP Reverse 2	GTTCCTGCTCAAAGAGCTCG	f/f: 404
	Deletion Reverse	CTGCAGCTCTGGATCTGAAG	WT: 1114<br>f/f: 262
Pgr$^{cre/+}$	Cre Forward 1	TATACCGATCTCCCTGGACG	WT: 302<br>Cre: 552
	Cre Forward 2	ATGTTTAGCTGGCCCAAATG	
	WT Reverse	CCCAAAGAGACACCAGGAAG	

Supplementary Table 4. Primer sequences for real-time RT-PCR

Gene		Sequence (5'-3')	Size (bp)
Ihh	Forward	CAGCAGTCTCTTCCATCCAGGAG	130
	Reverse	CGGTTCTGTCCATTCATCTTGTG	
Ptch1	Forward	GATGAACGCCTTTATGGTGTGGG	117
	Reverse	CGGTACTTGTAGTTGGGGTGGTC	
Ptch2	Forward	GCCGAGTATAGCTGCCTCAAGG	181
	Reverse	CGAACCGTGTTGGCTGGTTGG	
Gli1	Forward	CAGCTACATCGCCTACCTC	122
	Reverse	GTCGTTGCTGCTCACTGTGC	
Gli2	Forward	GCACTGGAGAAGAAAGAAGCC	164
	Reverse	CTCATGTCAATCGGCAAAGGCG	
Gli3	Forward	CACAGCTCTACGGCGACTG	168
	Reverse	CTGCATAGTGATTGCGTTTCTTC	
Gata2	Forward	CAGCAGTCTCTTCCATCCAGGAG	207
	Reverse	CGGTTCTGTCCATTCATCTTGTG	
Sox17	Forward	GATGAACGCCTTTATGGTGTGGG	201
	Reverse	CGGTACTTGTAGTTGGGGTGGTC	
Nr2f2	Forward	GCCGAGTATAGCTGCCTCAAGG	154
	Reverse	CGAACCGTGTTGGCTGGTTGG	
Hand2	Forward	CAGCTACATCGCCTACCTC	137
	Reverse	GTCGTTGCTGCTCACTGTGC	
Fgf2	Forward	CCACTTCAAGGACCCCAAGCG	267
	Reverse	TTGTAGTTATTAGATTCCAGTCG	
Fgf18	Forward	TTGGCAGCCGAGGAGAATGTGG	243
	Reverse	TTCTGTCTCCTTGCCCTTGATCC	
Cfp1	Forward	CTGGCGGTTCAGGTCCAAT	170
	Reverse	TTCCAGGCAATCCACGAGC	
Rpl7	Forward	TCAATGGAGTAAGCCCAAAG	246
	Reverse	CAAGAGACCGAGCAATCAAG	

Gene		Sequence (5'-3')	Size (bp)
CFP1	Forward	GTCGGGACATGAAGAAGTTCGGG	296
	Reverse	AGGTAGGTCCTCATCTGAGAGTGG	
GATA2	Forward	GGCTCGTTCCTGTTCAGAAG	104
	Reverse	CACAGGCATTGCACAGGTAG	
SOX17	Forward	GCTTTCATGGTGTGGGCTAAGG	189
	Reverse	CTTGTAGTTGGGGTGGTCCTGC	
IHH	Forward	CAGACATCATCTTCAAGGACGAGG	240
	Reverse	CCAGCAGTCCATACTTATTGCGG	
RPL19	Forward	TGAGACCAATGAAATCGCCAATGC	94
	Reverse	ATGGACCGTCACAGGCTTGC	

Supplementary Table 5. List of primary antibodies

Antibody	Host	Cat. no	Supplier	Dilution	Use for
CGBP (CFP1)	Rabbit	ab198977	Abcam	1:200,1:1500	IHC,IF,WB
Acetylated tubulin	Mouse	T7451	Sigma	1:500	IF
PGR	Rabbit	MA1-410	Thermo	1:200,1:1000	IF,WB
ESR	Rabbit	sc-542	Santa Cruz	1:200,1:1000	IF,WB
KI67	Rabbit	ab16667	Abcam	1:200	IF
GATA2	Rabbit	NBP1-82581	Novus	1:50	IHC
SOX17	Mouse	ab84990	Abcam	1:100	IHC
GAPDH	Rabbit	2118	Cell Signaling	1:2000	WB
CGBP (CFP1)	Rabbit	ab56035	Abcam	5 mg	ChIP, IP
H3K4me3	Rabbit	39159	Active motif	5 mg	ChIP
SET1	Rabbit	ab70378	Abcam	5 mg	ChIP
Normal rabbit IgG	Rabbit	sc-2027	Santa Cruz	5 mg	ChIP, IP

IHC, immunohistochemistry; IF, immunofluorescence; WB, western blotting; ChIP, chromatin immunoprecipitation

Supplementary Table 6. List of secondary antibodies

Target	Source	Conjugated	Cat. no	Supplier	Dilution	Use for
anti-rabbit IgG (H+L)	Goat	HRP	#31210	Invitrogen	1:200,1:3000	IHC, WB
anti-mouse Ig G(H+L)	Goat	HRP	NC11430KR	Thermo	1:200,1:3000	IHC, WB
anti-rabbit IgG (H+L)	Goat	Alexa Fluor 488	A-11008	Invitrogen	1:200	IF
anti-mouse Ig G(H+L)	Goat	Alexa Fluor 488	A-11001	Invitrogen	1:200	IF
anti-rabbit IgG (H+L)	Goat	Alexa Fluor 594	A-11012	Invitrogen	1:200	IF

HRP, horseradish peroxidase; IHC, immunohistochemistry; WB, western blotting; IF, /immunofluorescence
