# Supplementary material for: CFP1 governs uterine epigenetic landscapes to intervene in progesterone responses for uterine physiology and suppression of endometriosis
Source: Nat Commun. 2023 Jun 3;14:3220. doi: 10.1038/s41467-023-39008-0 (PMC10239508; doi:10.1038/s41467-023-39008-0)
Supplement: Supplementary file 3 — Description of Additional Supplementary Files [file 41467_2023_39008_MOESM3_ESM.pdf]

Description of Additional Supplementary Files

File Name: Supplementary Data 1
Description: H3K4me3-dependent *Cfp1* direct target genes

File Name: Supplementary Data 2
Description: H3K4me3-independent *Cfp1* direct target genes
