# Supplementary material for: CFP1 governs uterine epigenetic landscapes to intervene in progesterone responses for uterine physiology and suppression of endometriosis
Source: Nat Commun. 2023 Jun 3;14:3220. doi: 10.1038/s41467-023-39008-0 (PMC10239508; doi:10.1038/s41467-023-39008-0)
Supplement: Supplementary file 4 — Supplementary Data 1 and 2 [file 41467_2023_39008_MOESM4_ESM.pdf]

Supplementary Data 1. H3K4me3-dependent *Cfp1* direct target genes

Rank	Gene	Log2(FC)	-LOG10(p-value)
1	*Lrp2*	-8.38	3.30
2	*Meig1*	-6.64	1.81
3	*Fam189a1*	-6.16	3.59
4	*Ano2*	-5.80	2.16
5	*Gm11541*	-5.32	4.37
6	*Pla2g10*	-5.16	1.82
7	*Cyp1b1*	-5.06	2.36
8	*Coch*	-5.06	2.46
9	*Slc2a3*	-5.01	2.49
10	*Wnt11*	-4.92	3.15
11	*Aspg*	-4.57	3.00
12	*Tmem132e*	-4.47	3.03
13	*Acot7*	-4.44	2.19
14	*Grid2ip*	-4.41	3.55
15	*Slc13a5*	-4.16	1.57
16	*Apela*	-3.90	3.81
17	*Kcnt1*	-3.80	3.77
18	*Ihh*	-3.72	3.06
19	*Scn4b*	-3.64	1.87
20	*Gldc*	-3.57	1.97
21	*Ovgp1*	-3.49	2.14
22	*Col26a1*	-3.43	2.60
23	*Hopx*	-3.43	2.93
24	*Rgs2*	-3.40	5.94

Rank	Gene	Log2(FC)	-LOG10(p-value)
25	*Has2*	-3.37	2.63
26	*Kcnc3*	-3.27	4.76
27	*Shisa3*	-3.25	2.23
28	*Tnfrsf9*	-3.21	2.27
29	*F2r*	-3.21	4.53
30	*Ednrb*	-3.08	5.37
31	*Syn2*	-3.04	3.00
32	*Nptx1*	-3.01	2.76
33	*Rtn1*	-2.99	3.79
34	*Stra6*	-2.95	3.82
35	*6030408B16Rik*	-2.94	2.41
36	*Pcyt1b*	-2.94	2.82
37	*Tspan11*	-2.90	3.69
38	*Pfkfb3*	-2.88	2.43
39	*Ube2ql1*	-2.88	3.71
40	*Map7d2*	-2.86	2.53
41	*Hif3a*	-2.84	3.14
42	*Gulo*	-2.83	1.92
43	*Timp4*	-2.80	2.10
44	*Unc80*	-2.77	2.03
45	*Nog*	-2.77	2.30
46	*Aqp11*	-2.76	2.68
47	*Clcn5*	-2.75	2.05
48	*Prss12*	-2.75	4.01
49	*Gstm2*	-2.67	2.26

Rank	Gene	Log2(FC)	-LOG10(p-value)
50	*Grik1*	-2.67	3.43
51	*Ggt6*	-2.65	2.33
52	*Pcx*	-2.63	3.08
53	*Ptger3*	-2.63	5.48
54	*Slc4a4*	-2.63	2.45
55	*Sstr2*	-2.61	1.36
56	*Nkx6-1*	-2.60	1.66
57	*Col23a1*	-2.60	2.74
58	*Ccbe1*	-2.58	3.59
59	*Nalcn*	-2.57	2.99
60	*Tekt1*	-2.56	2.82
61	*Hrk*	-2.55	3.02
62	*Stac2*	-2.53	2.61
63	*Insl6*	-2.52	2.38
64	*Mest*	-2.43	2.95
65	*Hhipl1*	-2.42	3.93
66	*Osr1*	-2.40	3.72
67	*Slc10a6*	-2.39	3.38
68	*Jam2*	-2.38	2.99
69	*Asphd2*	-2.37	4.40
70	*Sfrp2*	-2.37	5.01
71	*Bdkrb2*	-2.36	3.11
72	*Tmem200c*	-2.35	2.58
73	*Cln5*	-2.35	6.21
74	*E330017L17Rik*	-2.34	1.62

Rank	Gene	Log2(FC)	-LOG10(p-value)
75	*Arvcf*	-2.34	2.16
76	*Vash2*	-2.33	3.30
77	*Sult1a1*	-2.29	4.07
78	*Gprc5b*	-2.28	3.07
79	*Kcnh1*	-2.24	1.64
80	*Thrsp*	-2.23	2.14
81	*Rims1*	-2.23	3.67
82	*Tmc7*	-2.22	1.97
83	*Kbtbd8*	-2.21	1.71
84	*Pthlh*	-2.20	3.41
85	*B930092H01Rik*	-2.18	2.18
86	*Wfdc1*	-2.18	3.72
87	*Sidt1*	-2.17	1.72
88	*Rdh1*	-2.16	1.62
89	*Rgs16*	-2.16	3.89
90	*Elovl2*	-2.12	2.18
91	*Plxnb1*	-2.11	3.88
92	*Adamts17*	-2.11	3.12
93	*Adamts20*	-2.08	1.88
94	*Rragd*	-2.07	2.88
95	*Myd88*	-2.06	2.99
96	*Fam167a*	-2.06	3.13
97	*Lrrn1*	-2.06	3.42
98	*Eppin*	-2.06	1.79
99	*Zfp57*	-2.04	4.95

Rank	Gene	Log2(FC)	-LOG10(p-value)
100	*Mthfd2*	-2.02	1.80
101	*Arl4d*	-2.02	3.57
102	*D630045J12Rik*	-2.01	4.13
103	*1810041L15Rik*	-2.01	3.09
104	*Nrcam*	-1.99	3.22
105	*Cd83*	-1.97	3.30
106	*BC051019*	-1.95	1.74
107	*Kcnk4*	-1.95	1.91
108	*Klf15*	-1.95	4.87
109	*Grin2b*	-1.94	1.83
110	*Trnp1*	-1.94	2.31
111	*Lin7a*	-1.92	2.85
112	*Zfp536*	-1.88	1.99
113	*Spock2*	-1.87	4.64
114	*Asl*	-1.86	2.80
115	*Nxpe3*	-1.85	3.26
116	*Gcnt4*	-1.84	4.65
117	*Medag*	-1.83	3.41
118	*Agtr1b*	-1.82	1.94
119	*Sybu*	-1.82	3.60
120	*Capn6*	-1.81	2.43
121	*Tppp*	-1.80	3.47
122	*Pdgfra*	-1.80	2.71
123	*Tmod2*	-1.79	3.90
124	*Nudt19*	-1.79	4.44

Rank	Gene	Log2(FC)	-LOG10(p-value)
125	*Fam46c*	-1.77	1.85
126	*Isg20*	-1.76	2.98
127	*Nsun7*	-1.76	3.13
128	*Etnk1*	-1.75	4.71
129	*Col18a1*	-1.74	4.43
130	*Camta1*	-1.73	4.85
131	*Ebpl*	-1.72	5.44
132	*Dhtkd1*	-1.72	2.93
133	*Fam69c*	-1.70	1.42
134	*Synpo2*	-1.70	3.44
135	*Chrna7*	-1.70	3.44
136	*Mfap5*	-1.70	3.56
137	*Vipr2*	-1.69	1.87
138	*Gata5*	-1.69	2.59
139	*Fam189a2*	-1.69	3.19
140	*D430019H16Rik*	-1.68	2.67
141	*Nckap5*	-1.68	2.76
142	*Sox5*	-1.68	3.29
143	*Brsk2*	-1.67	2.45
144	*Artn*	-1.67	3.41
145	*B230208H11Rik*	-1.66	1.88
146	*Unc5d*	-1.66	1.99
147	*Cfap46*	-1.66	2.41
148	*Penk*	-1.64	3.58
149	*Cldn3*	-1.63	2.17

Rank	Gene	Log2(FC)	-LOG10(p-value)
150	*Aldh1l1*	-1.63	2.85
151	*Scin*	-1.62	2.94
152	*Nol4*	-1.62	3.86
153	*Tsc22d3*	-1.61	3.35
154	*Usp2*	-1.60	3.87
155	*Ankrd29*	-1.60	2.23
156	*Foxa2*	-1.59	1.43
157	*Ccdc105*	-1.58	1.57
158	*Cnn3*	-1.58	3.76
159	*Fah*	-1.57	3.95
160	*Cpxm2*	-1.57	2.87
161	*C430049B03Rik*	-1.57	4.15
162	*Gap43*	-1.56	2.18
163	*Ccdc141*	-1.55	4.02
164	*Pacsin3*	-1.54	2.91
165	*Fam181b*	-1.54	2.73
166	*Ccdc13*	-1.54	1.66
167	*Muc2*	-1.54	1.69
168	*Trim63*	-1.54	2.23
169	*Ppm1e*	-1.54	3.35
170	*Olfml2b*	-1.54	4.22
171	*Pdgfd*	-1.53	2.12
172	*Cdc14a*	-1.53	2.42
173	*Hilpda*	-1.53	2.70
174	*Itpr2*	-1.52	3.65

Rank	Gene	Log2(FC)	-LOG10(p-value)
175	*Pygl*	-1.52	4.34
176	*Kctd14*	-1.51	2.07
177	*Twist2*	-1.49	3.06
178	*Mab21l3*	-1.49	1.71
179	*Vdr*	-1.48	1.90
180	*Mt3*	-1.47	1.70
181	*Igf1*	-1.47	3.38
182	*Piezo2*	-1.47	3.74
183	*Ammecr1*	-1.47	3.01
184	*Frmd5*	-1.46	1.49
185	*Slc16a11*	-1.46	2.51
186	*Spp1*	-1.45	1.62
187	*Rasl10b*	-1.45	1.65
188	*Kcnn2*	-1.44	2.26
189	*Pfn2*	-1.44	4.10
190	*Egfr*	-1.43	2.82
191	*Per1*	-1.43	5.17
192	*Adipor2*	-1.43	2.57
193	*Bambi*	-1.43	2.89
194	*Cyp27a1*	-1.42	2.58
195	*Tnfsf13b*	-1.42	2.78
196	*Cdh6*	-1.42	2.93
197	*Rassf2*	-1.41	3.06
198	*Klf2*	-1.41	3.13
199	*Gpr176*	-1.40	2.14

Rank	Gene	Log2(FC)	-LOG10(p-value)
200	*Them5*	-1.40	1.44
201	*Nde1*	-1.40	2.45
202	*Tbx2*	-1.40	2.90
203	*Tsku*	-1.39	2.12
204	*Dpysl4*	-1.39	3.50
205	*Procr*	-1.38	3.67
206	*Gdf11*	-1.38	3.21
207	*St6gal2*	-1.38	1.78
208	*Tgfbrap1*	-1.37	2.37
209	*Bdh2*	-1.37	2.95
210	*Fzd1*	-1.37	3.73
211	*Pamr1*	-1.37	1.98
212	*Spsb4*	-1.36	1.98
213	*Efcab12*	-1.36	2.57
214	*Il15ra*	-1.35	3.04
215	*Mtfp1*	-1.35	1.41
216	*Cacnb4*	-1.35	2.01
217	*2810410L24Rik*	-1.35	2.59
218	*Ebf1*	-1.35	3.12
219	*Slc1a1*	-1.34	2.52
220	*Lmtk3*	-1.34	2.60
221	*Thy1*	-1.34	3.06
222	*Sccpdh*	-1.34	1.80
223	*Nlgn3*	-1.33	2.77
224	*Sec14l5*	-1.32	1.32

Rank	Gene	Log2(FC)	-LOG10(p-value)
225	*Agpat4*	-1.32	3.05
226	*Slc39a14*	-1.32	3.40
227	*Vim*	-1.31	3.77
228	*Slc47a2*	-1.31	2.06
229	*Rnf157*	-1.31	2.43
230	*Clmn*	-1.30	2.83
231	*Bcl6b*	-1.30	3.71
232	*Gpr83*	-1.30	1.89
233	*C1qtnf6*	-1.30	3.25
234	*Sema6c*	-1.30	3.26
235	*Sema5a*	-1.29	2.37
236	*Fosb*	-1.29	2.07
237	*Antxr2*	-1.29	2.34
238	*Islr2*	-1.28	2.37
239	*Rln1*	-1.28	1.38
240	*Pianp*	-1.28	1.39
241	*Soat1*	-1.28	2.66
242	*Gm3636*	-1.28	3.12
243	*Ntng2*	-1.26	1.94
244	*Lrp4*	-1.26	2.62
245	*Car11*	-1.26	2.63
246	*Ggta1*	-1.26	3.41
247	*Adam11*	-1.25	3.49
248	*Prdm5*	-1.25	3.67
249	*Tmem178b*	-1.24	1.41

Rank	Gene	Log2(FC)	-LOG10(p-value)
250	*Creb5*	-1.24	2.08
251	*Mark1*	-1.23	3.04
252	*Dab1*	-1.23	2.41
253	*Gm3558*	-1.23	3.22
254	*Wt1*	-1.23	3.23
255	*Lactb2*	-1.22	2.22
256	*Kcnip1*	-1.22	2.46
257	*Stac*	-1.21	1.56
258	*Cecr2*	-1.21	2.09
259	*C2cd4c*	-1.20	1.32
260	*Fxyd4*	-1.20	1.69
261	*Frmd6*	-1.19	3.98
262	*Slc2a12*	-1.19	2.09
263	*Scara3*	-1.19	2.45
264	*Sema6a*	-1.19	2.80
265	*Gm3696*	-1.18	2.43
266	*Chsy3*	-1.18	2.55
267	*Heyl*	-1.18	3.21
268	*Lurap1*	-1.18	2.15
269	*Nrgn*	-1.18	2.61
270	*Snhg5*	-1.18	3.51
271	*Gm9895*	-1.17	1.52
272	*Ptpro*	-1.16	2.87
273	*Nr5a2*	-1.16	3.38
274	*Gli3*	-1.15	2.35

Rank	Gene	Log2(FC)	-LOG10(p-value)
275	*Cacna1e*	-1.14	2.08
276	*Fblim1*	-1.14	3.56
277	*Gjc1*	-1.14	3.65
278	*Rasl12*	-1.14	3.66
279	*Gm11346*	-1.14	2.99
280	*Kcnd3*	-1.14	1.90
281	*Gcnt1*	-1.14	2.22
282	*Osgin2*	-1.13	2.14
283	*Galnt18*	-1.13	2.96
284	*Peli2*	-1.13	3.08
285	*Faxc*	-1.13	3.40
286	*Zhx3*	-1.13	3.81
287	*Abat*	-1.13	3.05
288	*Rel*	-1.12	3.34
289	*Usp31*	-1.12	3.67
290	*Fbxo41*	-1.12	1.59
291	*Slc26a6*	-1.11	1.77
292	*Gabbr1*	-1.11	2.93
293	*Nkain4*	-1.11	1.77
294	*Chst2*	-1.11	2.98
295	*Runx1t1*	-1.11	2.22
296	*Arhgap20*	-1.11	2.22
297	*AF529169*	-1.10	2.97
298	*Gm5141*	-1.10	2.42
299	*Gm9833*	-1.09	2.47

Rank	Gene	Log2(FC)	-LOG10(p-value)
300	*Rab40b*	-1.09	2.58
301	*Fam212b*	-1.08	1.92
302	*Rcor2*	-1.07	2.50
303	*Pde7a*	-1.07	4.01
304	*Pagr1a*	-1.07	4.94
305	*Gxylt2*	-1.06	2.40
306	*Snord104*	-1.06	1.38
307	*Ch25h*	-1.06	2.13
308	*Fbxo10*	-1.06	2.52
309	*Rab39b*	-1.05	1.78
310	*Ssfa2*	-1.05	3.92
311	*Nos2*	-1.05	1.61
312	*Dcn*	-1.05	1.81
313	*Aldh1l2*	-1.05	1.90
314	*Pus7*	-1.05	2.62
315	*Ggt7*	-1.04	2.19
316	*Ubxn11*	-1.04	3.07
317	*Gm29683*	-1.04	3.08
318	*Doc2g*	-1.04	3.43
319	*Zfp566*	-1.03	3.05
320	*Fam167b*	-1.02	1.54
321	*6530402F18Rik*	-1.02	1.84
322	*Svep1*	-1.02	2.45
323	*Fam126a*	-1.02	2.65
324	*Nyap1*	-1.02	2.96

Rank	Gene	Log2(FC)	-LOG10(p-value)
325	*Mpp3*	-1.02	4.21
326	*Ubn1*	-1.02	2.65
327	*Celf4*	-1.02	1.52
328	*Prune2*	-1.02	2.68
329	*Tesk2*	-1.02	3.28
330	*Cabp1*	-1.01	2.11
331	*Sh3bp1*	-1.01	2.08
332	*Msi1*	-1.01	2.59
333	*Sulf1*	-1.01	1.95
334	*Chrd*	-1.01	2.21
335	*Ccdc74a*	-1.01	2.67
336	*Ccdc30*	-1.00	2.29
337	*Ank2*	-1.00	2.56
338	*Gramd3*	-1.00	1.64
339	*Wnt2b*	-1.00	2.50
340	*Pde1a*	-1.00	2.56
341	*Tada2a*	-0.99	2.44
342	*Shc3*	-0.99	3.03
343	*Rps6ka2*	-0.99	3.08
344	*Rarres2*	-0.99	2.99
345	*Nova2*	-0.99	1.90
346	*Kif17*	-0.99	2.37
347	*Sh3bgr*	-0.99	2.73
348	*1700067K01Rik*	-0.99	2.05
349	*Zfp647*	-0.99	2.80

Rank	Gene	Log2(FC)	-LOG10(p-value)
350	*Fbxo17*	-0.99	3.76
351	*2810025M15Rik*	-0.98	2.41
352	*Gm10560*	-0.98	2.09
353	*Gem*	-0.98	2.09
354	*Fsd1*	-0.97	1.90
355	*Lpar1*	-0.97	4.91
356	*Rhpn1*	-0.97	1.76
357	*Pnpt1*	-0.97	4.82
358	*Cyp46a1*	-0.96	1.35
359	*Fads2*	-0.96	1.69
360	*2810468N07Rik*	-0.96	1.78
361	*Slc22a23*	-0.95	2.02
362	*Cbr3*	-0.95	1.76
363	*Cpeb1*	-0.95	2.30
364	*Zbtb20*	-0.95	1.80
365	*Pvt1*	-0.95	2.50
366	*Mertk*	-0.95	2.59
367	*Gja5*	-0.95	2.81
368	*Shc2*	-0.95	3.52
369	*Them6*	-0.95	2.52
370	*Matn2*	-0.94	2.11
371	*Add3*	-0.94	3.07
372	*Iah1*	-0.94	3.31
373	*Msrb1*	-0.94	2.33
374	*Jag2*	-0.93	2.85

Rank	Gene	Log2(FC)	-LOG10(p-value)
375	*1700017B05Rik*	-0.93	3.25
376	*Avl9*	-0.92	2.19
377	*Ric1*	-0.92	2.87
378	*S100a10*	-0.92	3.15
379	*4931440P22Rik*	-0.92	3.74
380	*Abcc4*	-0.92	3.57
381	*Pcdh19*	-0.92	3.92
382	*Gpc1*	-0.92	1.76
383	*Cela1*	-0.92	1.85
384	*Fam149a*	-0.92	3.03
385	*Golga5*	-0.92	2.64
386	*Podxl*	-0.92	2.88
387	*Acot2*	-0.91	1.31
388	*Prtg*	-0.91	1.82
389	*Ica1l*	-0.91	2.29
390	*Rgs9*	-0.91	2.71
391	*Gramd1a*	-0.91	4.74
392	*Rhebl1*	-0.91	1.75
393	*Flna*	-0.90	2.83
394	*Gpt2*	-0.90	2.13
395	*Ppara*	-0.90	2.42
396	*Tnik*	-0.89	3.31
397	*Adcy9*	-0.89	2.93
398	*Rnd1*	-0.89	1.36
399	*E130308A19Rik*	-0.89	2.57

Rank	Gene	Log2(FC)	-LOG10(p-value)
400	*Smox*	-0.88	1.72
401	*Dock5*	-0.88	2.05
402	*Tgif2*	-0.88	3.07
403	*Gpc2*	-0.88	3.52
404	*Adra2a*	-0.88	3.94
405	*Cldn4*	-0.88	1.65
406	*Tcea2*	-0.88	2.74
407	*Fuk*	-0.88	1.55
408	*Jph4*	-0.88	2.30
409	*Apold1*	-0.88	3.70
410	*Slc4a7*	-0.88	2.57
411	*Slc29a1*	-0.87	2.67
412	*Slc7a6*	-0.87	2.78
413	*Smpd5*	-0.87	1.75
414	*Metap1d*	-0.87	1.87
415	*Rfx2*	-0.87	2.07
416	*Fbln7*	-0.87	2.15
417	*Dll1*	-0.86	2.16
418	*Rlf*	-0.86	2.43
419	*Tppp3*	-0.86	2.53
420	*Egflam*	-0.86	3.42
421	*Srm*	-0.86	1.71
422	*Adrb1*	-0.86	1.89
423	*Zfp691*	-0.85	1.37
424	*Simc1*	-0.85	2.93

Rank	Gene	Log2(FC)	-LOG10(p-value)
425	*Lama2*	-0.85	2.18
426	*Aff3*	-0.85	1.37
427	*Zfp956*	-0.85	1.76
428	*Gpr150*	-0.85	1.37
429	*Ccdc106*	-0.85	2.27
430	*Rnf144b*	-0.84	2.15
431	*S100a6*	-0.84	2.21
432	*Zfp454*	-0.84	2.58
433	*Zfp275*	-0.84	2.64
434	*Lgals1*	-0.84	1.63
435	*Rnf182*	-0.84	1.89
436	*Ptgfr*	-0.84	1.32
437	*Ap3b2*	-0.84	1.43
438	*Rasip1*	-0.83	2.77
439	*Atp8b5*	-0.83	1.51
440	*Zfp30*	-0.83	2.51
441	*Cdk5r1*	-0.83	3.13
442	*Fendrr*	-0.83	2.43
443	*Nid1*	-0.83	3.11
444	*Id3*	-0.83	1.99
445	*Slc7a10*	-0.83	2.69
446	*Runx2*	-0.83	2.82
447	*Tgfbr3*	-0.82	1.60
448	*9330159M07Rik*	-0.82	2.21
449	*Ddit4l*	-0.82	2.07

Rank	Gene	Log2(FC)	-LOG10(p-value)
450	*Sobp*	-0.82	2.88
451	*Ppp1r12c*	-0.82	3.50
452	*Fez1*	-0.82	2.59
453	*Maf*	-0.81	2.00
454	*Hmgn3*	-0.81	3.12
455	*Notch3*	-0.81	3.59
456	*Prob1*	-0.81	1.81
457	*6030419C18Rik*	-0.81	2.01
458	*Arap1*	-0.81	2.32
459	*Fastkd1*	-0.80	2.39
460	*Itih5*	-0.80	2.00
461	*Kif27*	-0.80	2.04
462	*Meis3*	-0.80	2.08
463	*Xylt1*	-0.80	2.09
464	*Tfap4*	-0.80	2.46
465	*Spats2*	-0.80	2.87
466	*Aldh6a1*	-0.79	2.61
467	*Lims2*	-0.79	1.82
468	*Bsdc1*	-0.79	2.14
469	*Arhgef37*	-0.79	3.84
470	*Mterf2*	-0.79	1.73
471	*Kctd6*	-0.79	2.21
472	*Mlxipl*	-0.79	2.40
473	*Maoa*	-0.79	2.78
474	*Fn1*	-0.79	2.88

Rank	Gene	Log2(FC)	-LOG10(p-value)
475	*S1pr1*	-0.79	2.95
476	*Myl9*	-0.78	2.45
477	*Uhrf1bp1l*	-0.78	4.67
478	*Btbd3*	-0.78	3.76
479	*Satb1*	-0.77	2.30
480	*Zfp961*	-0.77	3.90
481	*Ankmy1*	-0.77	1.65
482	*Slc2a13*	-0.77	2.43
483	*Hacd3*	-0.77	2.44
484	*Tekt2*	-0.77	2.71
485	*Tpm1*	-0.77	2.96
486	*St6galnac4*	-0.76	2.18
487	*Tmem120b*	-0.76	2.40
488	*Thsd1*	-0.76	2.27
489	*C1qtnf4*	-0.76	1.60
490	*2700038G22Rik*	-0.76	1.68
491	*Inpp4a*	-0.76	2.61
492	*Doc2a*	-0.76	3.23
493	*Gpc3*	-0.76	4.17
494	*Nedd4l*	-0.76	2.39
495	*Klf8*	-0.76	2.45
496	*Ece2*	-0.75	2.83
497	*Atxn7l2*	-0.75	2.83
498	*Kcnip3*	-0.75	2.40
499	*Bahcc1*	-0.75	2.56

Rank	Gene	Log2(FC)	-LOG10(p-value)
500	*Sipa1*	-0.75	3.11
501	*St6galnac3*	-0.75	2.40
502	*Klhl8*	-0.74	1.31
503	*Zfp583*	-0.74	1.76
504	*Egr3*	-0.74	1.76
505	*9130019P16Rik*	-0.74	1.78
506	*Slc1a4*	-0.74	1.96
507	*Impdh1*	-0.74	1.97
508	*Smtn*	-0.74	2.42
509	*Mex3a*	-0.74	2.73
510	*Gimap9*	-0.74	2.73
511	*Map3k3*	-0.74	3.50
512	*Col7a1*	-0.74	1.36
513	*Slc25a29*	-0.74	2.68
514	*Rab26*	-0.74	1.84
515	*Dhh*	-0.74	2.10
516	*Lrp3*	-0.74	2.20
517	*Nlk*	-0.74	2.98
518	*Tmem65*	-0.74	2.99
519	*Dpf1*	-0.73	2.46
520	*Rnase4*	-0.73	1.95
521	*Aldh1b1*	-0.73	2.27
522	*Cacna1d*	-0.73	2.63
523	*Rogdi*	-0.73	2.64
524	*Hey2*	-0.73	2.48

Rank	Gene	Log2(FC)	-LOG10(p-value)
525	*Arid3b*	-0.73	1.96
526	*Elmo1*	-0.73	2.34
527	*Hspa12b*	-0.73	2.85
528	*Dctd*	-0.73	3.77
529	*Tmcc2*	-0.72	2.46
530	*Gabrb2*	-0.72	1.40
531	*6720489N17Rik*	-0.72	1.43
532	*Arhgap28*	-0.72	2.27
533	*Vldlr*	-0.72	2.30
534	*4930592I03Rik*	-0.72	1.44
535	*Jph1*	-0.72	2.35
536	*Rce1*	-0.72	3.01
537	*Tcp11*	-0.72	1.63
538	*Bnip3*	-0.72	2.11
539	*Mmp23*	-0.72	2.15
540	*Smarca2*	-0.72	2.46
541	*Arhgef25*	-0.72	3.55
542	*Tmem218*	-0.72	1.33
543	*Sfmbt2*	-0.72	1.65
544	*Myrip*	-0.72	1.83
545	*Tmem231*	-0.72	2.37
546	*Tacc1*	-0.71	2.50
547	*Enpp3*	-0.71	1.43
548	*Nr6a1*	-0.71	1.73
549	*Dclk2*	-0.71	2.11

Rank	Gene	Log2(FC)	-LOG10(p-value)
550	*Mllt3*	-0.71	2.53
551	*She*	-0.71	4.00
552	*Gnal*	-0.71	1.69
553	*Pmp22*	-0.71	3.06
554	*Zfp74*	-0.70	2.02
555	*Slc25a22*	-0.70	2.15
556	*Slc37a4*	-0.70	2.28
557	*Rpl22l1*	-0.70	3.76
558	*Tmem150b*	-0.70	1.41
559	*Stx1a*	-0.70	3.63
560	*Zfp811*	-0.70	1.37
561	*Lrrc61*	-0.70	1.40
562	*Aif1l*	-0.69	1.67
563	*Sec23b*	-0.69	1.42
564	*Olfml3*	-0.69	1.48
565	*Herc1*	-0.69	2.85
566	*Ntn4*	-0.69	1.56
567	*Tle4*	-0.69	1.64
568	*Zfp532*	-0.69	3.43
569	*Lrp12*	-0.69	1.66
570	*Ikzf1*	-0.69	3.95
571	*Pgrmc2*	-0.69	3.97
572	*Fndc5*	-0.69	1.37
573	*Pygo1*	-0.69	1.38
574	*Sesn1*	-0.69	2.04

Rank	Gene	Log2(FC)	-LOG10(p-value)
575	*Bfar*	-0.69	2.40
576	*Frs2*	-0.68	2.79
577	*Plekha5*	-0.68	2.01
578	*Pdp2*	-0.68	2.11
579	*Dusp7*	-0.68	2.18
580	*2510009E07Rik*	-0.68	2.20
581	*Gnb4*	-0.68	2.79
582	*Grin2d*	-0.68	1.80
583	*Ndrg4*	-0.68	3.76
584	*Mical2*	-0.67	2.01
585	*Atf5*	-0.67	2.85
586	*Gm10941*	-0.67	1.67
587	*Sox12*	-0.67	3.10
588	*Alg13*	-0.67	1.80
589	*Igdcc4*	-0.67	2.12
590	*Sorbs3*	-0.67	2.45
591	*Dpysl3*	-0.67	2.71
592	*Fbln5*	-0.67	2.35
593	*Adh5*	-0.66	1.82
594	*Ankrd13b*	-0.66	1.93
595	*Sec61a2*	-0.66	2.51
596	*Cep68*	-0.66	2.79
597	*Ankrd10*	-0.66	3.95
598	*Zkscan4*	-0.66	1.74
599	*Ctxn1*	-0.66	2.72

Rank	Gene	Log2(FC)	-LOG10(p-value)
600	*C1s1*	-0.65	1.64
601	*Prpf40b*	-0.65	1.95
602	*Zfpm2*	-0.65	1.39
603	*Eml1*	-0.65	2.49
604	*Rhbdl3*	-0.65	1.56
605	*Clip3*	-0.65	1.63
606	*Cyr61*	-0.65	1.92
607	*Rabep1*	-0.65	1.93
608	*Col4a2*	-0.64	2.02
609	*Neo1*	-0.64	2.03
610	*Fkbp1a*	-0.64	2.27
611	*Snora65*	-0.64	2.34
612	*Lamc1*	-0.64	2.12
613	*Adcy3*	-0.64	2.58
614	*Aplp1*	-0.64	1.40
615	*Zfyve16*	-0.64	3.25
616	*Itpk1*	-0.63	1.97
617	*Gm16596*	-0.63	1.84
618	*Flot2*	-0.63	2.34
619	*Eid2*	-0.63	2.44
620	*Dixdc1*	-0.63	1.38
621	*Sec16b*	-0.63	2.03
622	*Ppp1r14a*	-0.63	2.26
623	*Zfp251*	-0.63	2.36
624	*Mpi*	-0.63	2.40

Rank	Gene	Log2(FC)	-LOG10(p-value)
625	*Fancc*	-0.63	2.65
626	*Gata2*	-0.63	1.32
627	*4933404O12Rik*	-0.63	2.11
628	*Zfp882*	-0.63	2.12
629	*Bckdhb*	-0.63	2.20
630	*Myadm*	-0.63	3.06
631	*Filip1*	-0.62	1.64
632	*Amotl2*	-0.62	2.24
633	*Pced1a*	-0.62	2.07
634	*Usp53*	-0.62	1.34
635	*Timp3*	-0.62	1.76
636	*Cdc42se2*	-0.62	2.88
637	*P4htm*	-0.62	2.61
638	*Dhrs11*	-0.62	2.74
639	*Mypop*	-0.61	2.79
640	*Snord55*	-0.61	3.26
641	*Lims1*	-0.61	2.47
642	*Zfp516*	-0.61	2.74
643	*Cbx2*	-0.61	1.41
644	*2010009K17Rik*	-0.61	1.81
645	*Hspa12a*	-0.61	2.02
646	*Tex9*	-0.61	2.30
647	*Hoxd11*	-0.61	2.34
648	*Ppp1r12b*	-0.61	2.35
649	*Pls3*	-0.61	2.70

Rank	Gene	Log2(FC)	-LOG10(p-value)
650	*Dimt1*	-0.61	4.14
651	*Casz1*	-0.61	1.94
652	*Dact1*	-0.61	2.04
653	*Zcchc18*	-0.61	2.09
654	*Ehd2*	-0.61	2.66
655	*Bche*	-0.60	1.64
656	*Arl4a*	-0.60	2.20
657	*Metap1*	-0.60	1.90
658	*Ccdc85b*	-0.60	1.96
659	*Mier3*	-0.60	2.65
660	*Amotl1*	-0.60	3.73
661	*Chn2*	-0.60	1.83
662	*Ddx58*	-0.60	2.47
663	*Gm11627*	-0.60	1.76
664	*Fgfr3*	-0.60	1.89
665	*Gstz1*	-0.59	1.97
666	*Spin2c*	-0.59	2.34
667	*Pdzd4*	-0.59	3.13
668	*Mtx3*	-0.59	2.05
669	*Pde4a*	-0.59	2.39
670	*Sh3rf1*	-0.59	1.41
671	*Pbx3*	-0.59	1.56
672	*Nrn1*	-0.58	2.38
673	*Cep97*	-0.58	2.54

Supplementary Data 2. H3K4me3-independent *Cfp1* direct target genes

Rank	Gene	Log2(FC)	-LOG10(p-value)
1	*Areg*	-7.64	1.43
2	*Rims3*	-7.16	2.14
3	*Olah*	-6.97	1.51
4	*Calb2*	-6.27	2.84
5	*Drd4*	-5.97	2.38
6	*Cyp3a25*	-5.44	1.96
7	*Slc5a11*	-5.32	2.68
8	*St8sia5*	-5.01	2.07
9	*Pga5*	-4.92	1.42
10	*Gzme*	-4.92	4.39
11	*9530059O14Rik*	-4.76	2.27
12	*Car4*	-4.64	2.49
13	*Frmpd1*	-4.64	3.16
14	*Lipf*	-4.47	1.59
15	*Sbspon*	-4.47	3.06
16	*Tmco2*	-4.44	5.13
17	*Kcnc4*	-4.41	2.18
18	*Wfdc3*	-4.38	1.72
19	*Epha8*	-4.35	3.08
20	*Serpinb12*	-3.99	1.62
21	*Dlg2*	-3.97	3.65
22	*Sval2*	-3.92	4.09
23	*Tex21*	-3.90	1.30
24	*Abcb5*	-3.90	2.36

Rank	Gene	Log2(FC)	-LOG10(p-value)
25	*Nme8*	-3.84	1.59
26	*Pdzrn4*	-3.84	2.15
27	*Fbxw14*	-3.78	4.11
28	*Sct*	-3.72	2.72
29	*Pkdcc*	-3.64	3.71
30	*Acbd7*	-3.59	1.73
31	*Bsnd*	-3.54	1.97
32	*Klrg1*	-3.46	2.00
33	*D430036J16Rik*	-3.43	1.66
34	*E130008D07Rik*	-3.43	2.56
35	*Lrriq4*	-3.40	1.96
36	*Cyp26c1*	-3.31	1.77
37	*Sult1d1*	-3.31	3.28
38	*Vgll3*	-3.27	2.27
39	*Gm38415*	-3.25	2.53
40	*2400006E01Rik*	-3.22	1.79
41	*Tdo2*	-3.20	2.83
42	*Car3*	-3.16	1.89
43	*Col24a1*	-3.15	3.99
44	*Has2os*	-3.04	1.98
45	*Cldn9*	-3.04	3.20
46	*Cyp4f15*	-3.01	2.38
47	*Pou5f1*	-2.97	1.32
48	*Ctsd*	-2.97	3.97
49	*Hrh3*	-2.94	1.74

Rank	Gene	Log2(FC)	-LOG10(p-value)
50	*Fkbp5*	-2.88	3.60
51	*Calb1*	-2.84	2.64
52	*Tex15*	-2.82	3.82
53	*Slco4c1*	-2.81	1.91
54	*Cyp3a57*	-2.81	3.05
55	*Gm8633*	-2.78	1.34
56	*Asb12*	-2.76	3.08
57	*Dsg4*	-2.73	1.45
58	*Cyp2j5*	-2.70	1.36
59	*Crp*	-2.63	2.94
60	*Aox4*	-2.63	4.84
61	*Fbxw27*	-2.63	2.21
62	*Map2*	-2.62	2.70
63	*Cd36*	-2.60	1.74
64	*Acta1*	-2.56	2.98
65	*Krt23*	-2.55	2.33
66	*Hs6st3*	-2.54	2.00
67	*Slc22a12*	-2.53	2.28
68	*Fbxw26*	-2.51	2.06
69	*Cdkn1c*	-2.51	5.48
70	*Sdr16c6*	-2.49	1.98
71	*Ctgf*	-2.45	4.79
72	*Kcnab1*	-2.43	2.78
73	*Lgi1*	-2.39	2.12
74	*Amhr2*	-2.37	2.88

Rank	Gene	Log2(FC)	-LOG10(p-value)
75	*Gm3985*	-2.35	2.38
76	*Rdh9*	-2.34	1.73
77	*Cutal*	-2.34	2.22
78	*Ccr3*	-2.31	1.67
79	*2310069G16Rik*	-2.30	2.93
80	*Cntnap5c*	-2.29	3.43
81	*Serpina3h*	-2.29	1.41
82	*Slc25a48*	-2.29	2.79
83	*Tex11*	-2.27	2.95
84	*Krt20*	-2.27	2.44
85	*8430426J06Rik*	-2.27	3.83
86	*Mt1*	-2.26	2.85
87	*AI507597*	-2.24	3.02
88	*Clca2*	-2.23	3.84
89	*Acan*	-2.19	1.96
90	*Apln*	-2.19	2.28
91	*Hoxa11*	-2.18	3.85
92	*Kcnb2*	-2.17	3.09
93	*St8sia2*	-2.17	3.24
94	*Prnd*	-2.16	3.23
95	*Efemp1*	-2.13	2.77
96	*Sox2*	-2.11	2.02
97	*Ptch1*	-2.10	2.38
98	*Fbxw18*	-2.10	1.66
99	*A330015K06Rik*	-2.09	2.37

Rank	Gene	Log2(FC)	-LOG10(p-value)
100	*Fam57b*	-2.08	2.35
101	*Lancl3*	-2.05	2.40
102	*4933405O20Rik*	-2.04	2.01
103	*S100a7a*	-2.04	2.81
104	*Tmed6*	-2.02	1.42
105	*Slain1*	-2.02	1.89
106	*Cldn1*	-2.02	2.89
107	*Spdef*	-1.99	1.83
108	*Klkb1*	-1.98	1.81
109	*Cntn3*	-1.95	2.16
110	*Mlc1*	-1.93	2.02
111	*Panct2*	-1.93	2.17
112	*Alox12e*	-1.93	2.84
113	*Slc39a4*	-1.93	3.71
114	*Slain1os*	-1.91	2.17
115	*Errfi1*	-1.91	4.68
116	*Hoxa10*	-1.90	5.43
117	*Gucy2c*	-1.89	1.80
118	*Dpep1*	-1.88	2.22
119	*Sh3tc2*	-1.88	2.53
120	*Gm38425*	-1.88	2.67
121	*Cpsf4l*	-1.82	1.83
122	*Gstm1*	-1.81	2.48
123	*Ncr1*	-1.80	1.32
124	*Scn3a*	-1.79	5.94

Rank	Gene	Log2(FC)	-LOG10(p-value)
125	*Pcp4*	-1.79	3.69
126	*Stk32b*	-1.75	1.74
127	*Srrm3*	-1.73	1.88
128	*Hhatl*	-1.73	2.55
129	*C9*	-1.73	2.38
130	*Lrfn2*	-1.72	1.65
131	*Draxin*	-1.71	1.84
132	*Adam12*	-1.71	3.89
133	*Gad1*	-1.70	1.36
134	*Otof*	-1.70	1.78
135	*1700001K23Rik*	-1.68	1.95
136	*Znhit6*	-1.68	3.11
137	*Gm13889*	-1.68	4.44
138	*Gdf7*	-1.65	2.35
139	*Postn*	-1.65	2.75
140	*Ly75*	-1.65	2.95
141	*Htr3b*	-1.63	1.59
142	*Ankfn1*	-1.63	1.88
143	*Gpx2*	-1.62	2.55
144	*Basp1*	-1.61	3.17
145	*Nfil3*	-1.60	3.37
146	*Snai1*	-1.57	3.06
147	*Gsg1*	-1.56	1.64
148	*Slc6a14*	-1.55	1.51
149	*Zfp239*	-1.55	4.46

Rank	Gene	Log2(FC)	-LOG10(p-value)
150	*B4galnt4*	-1.54	2.33
151	*Col9a2*	-1.53	1.43
152	*Ap1s3*	-1.52	2.23
153	*Nptx2*	-1.52	2.38
154	*Entpd2*	-1.52	2.93
155	*Apoc2*	-1.52	1.40
156	*Nav3*	-1.51	3.21
157	*Dapl1*	-1.51	2.31
158	*Neu2*	-1.50	2.55
159	*Kcnip2*	-1.50	1.78
160	*B3gnt7*	-1.50	2.11
161	*Ntrk3*	-1.50	2.57
162	*Lipc*	-1.49	2.43
163	*Tmem132cos*	-1.49	2.64
164	*Kbtbd11*	-1.49	3.82
165	*A330093E20Rik*	-1.49	2.06
166	*Gm3383*	-1.49	2.85
167	*Rgs5*	-1.47	1.89
168	*Alpk2*	-1.45	1.43
169	*Nme4*	-1.45	4.31
170	*Actg2*	-1.45	2.09
171	*Fscn1*	-1.45	3.98
172	*Arg2*	-1.44	3.23
173	*Ms4a4d*	-1.43	1.63
174	*Rnase2b*	-1.43	4.59

Rank	Gene	Log2(FC)	-LOG10(p-value)
175	*1700025B11Rik*	-1.43	4.59
176	*Sorl1*	-1.43	3.19
177	*Hsd11b2*	-1.43	3.25
178	*Higd1b*	-1.42	1.92
179	*Sertm1*	-1.42	1.60
180	*Prr5l*	-1.40	1.51
181	*Gm11545*	-1.40	2.56
182	*1700110K17Rik*	-1.40	4.17
183	*Gfi1*	-1.39	1.48
184	*Dtx4*	-1.38	3.13
185	*Cspg5*	-1.37	1.91
186	*Fam186b*	-1.37	2.84
187	*Rnf223*	-1.37	3.18
188	*Clca3a1*	-1.36	1.94
189	*2610042L04Rik*	-1.36	2.25
190	*Megf10*	-1.36	2.27
191	*Amer2*	-1.35	1.86
192	*Nefh*	-1.35	1.53
193	*Map3k13*	-1.34	2.19
194	*Hspb8*	-1.34	2.70
195	*Stmn3*	-1.33	1.79
196	*Ldhd*	-1.33	3.47
197	*C630043F03Rik*	-1.33	2.64
198	*Gm21119*	-1.33	1.87
199	*Col15a1*	-1.31	2.22

Rank	Gene	Log2(FC)	-LOG10(p-value)
200	*Inha*	-1.31	2.13
201	*Grb10*	-1.31	2.94
202	*H2-M10.2*	-1.31	1.57
203	*Nkain1*	-1.30	2.63
204	*Gm3488*	-1.30	2.64
205	*Mkrn3*	-1.30	1.75
206	*Fgfr2*	-1.30	2.96
207	*Sertad4*	-1.29	4.17
208	*Gm2042*	-1.29	1.64
209	*Gm3317*	-1.29	2.51
210	*Rorb*	-1.29	2.87
211	*LOC100861615*	-1.28	3.03
212	*Tiparp*	-1.28	4.47
213	*Fam43a*	-1.28	2.28
214	*Bcorl1*	-1.27	3.08
215	*Gja10*	-1.27	2.24
216	*Cpz*	-1.27	3.53
217	*Lbp*	-1.27	2.26
218	*Dnm3os*	-1.27	2.60
219	*Klf9*	-1.26	2.86
220	*1700016D06Rik*	-1.26	1.39
221	*C630031E19Rik*	-1.25	1.53
222	*Lhx6*	-1.25	1.62
223	*Lbx2*	-1.24	2.37
224	*Ak4*	-1.23	2.55

Rank	Gene	Log2(FC)	-LOG10(p-value)
225	*Rarres1*	-1.23	1.54
226	*Cd177*	-1.23	2.59
227	*Htr2a*	-1.23	3.49
228	*Adora2b*	-1.22	2.05
229	*Tnxb*	-1.22	2.07
230	*Tshz3*	-1.21	2.76
231	*Il4*	-1.20	2.59
232	*1700093K21Rik*	-1.20	1.63
233	*Lamc3*	-1.20	2.98
234	*Gm3264*	-1.19	2.48
235	*Asic3*	-1.19	2.25
236	*Sag*	-1.18	2.27
237	*Hook1*	-1.18	2.44
238	*Nxpe2*	-1.17	3.17
239	*Rnf150*	-1.17	2.66
240	*Nrxn2*	-1.16	2.79
241	*Apbb3*	-1.16	2.11
242	*Kctd12*	-1.15	3.05
243	*C230035I16Rik*	-1.15	1.88
244	*Npas4*	-1.14	2.55
245	*Cd93*	-1.14	3.71
246	*Psapl1*	-1.14	2.08
247	*Gas5*	-1.11	3.32
248	*Srrm4os*	-1.11	2.34
249	*Snord12*	-1.10	2.05

Rank	Gene	Log2(FC)	-LOG10(p-value)
250	*Lcat*	-1.10	1.46
251	*Asic4*	-1.10	1.76
252	*Ogdhl*	-1.10	3.23
253	*Miat*	-1.09	1.77
254	*Hemgn*	-1.09	1.41
255	*Gpr4*	-1.09	2.27
256	*Myh11*	-1.09	3.23
257	*Atp10a*	-1.08	3.32
258	*Tle2*	-1.08	2.04
259	*Sept4*	-1.08	2.26
260	*Cyp2t4*	-1.06	2.08
261	*Snhg12*	-1.06	2.31
262	*Gm4832*	-1.06	1.64
263	*4921517D22Rik*	-1.05	1.38
264	*Snord14a*	-1.05	1.71
265	*Vsig2*	-1.05	2.27
266	*Irf6*	-1.04	1.82
267	*Sox17*	-1.04	2.03
268	*Fam78a*	-1.04	2.23
269	*Boc*	-1.04	3.01
270	*Kcng1*	-1.04	2.02
271	*Gm10409*	-1.03	2.85
272	*Prdm1*	-1.03	2.55
273	*Rtn4rl2*	-1.02	1.95
274	*Snora61*	-1.02	1.97

Rank	Gene	Log2(FC)	-LOG10(p-value)
275	*Cd200*	-1.02	3.30
276	*Morc4*	-1.01	3.05
277	*4930447N08Rik*	-1.01	1.80
278	*Il15*	-1.00	1.84
279	*Nr2f2*	-1.00	2.95
280	*Gm3020*	-1.00	2.22
281	*Usp50*	-0.99	1.76
282	*Mfhas1*	-0.98	3.27
283	*Myc*	-0.97	4.26
284	*Smok4a*	-0.97	1.37
285	*Rbfox3*	-0.97	1.90
286	*Prr33*	-0.97	1.98
287	*Ankrd33b*	-0.97	2.01
288	*Ccdc92*	-0.96	1.99
289	*Nt5dc2*	-0.95	1.80
290	*Lrrtm1*	-0.95	1.99
291	*Hoxd10*	-0.95	3.11
292	*Sfi1*	-0.95	2.24
293	*Marcksl1*	-0.94	2.50
294	*Ccdc85a*	-0.94	1.51
295	*Hba-a1*	-0.94	1.61
296	*Fam129c*	-0.93	2.02
297	*Adgrd1*	-0.92	1.88
298	*Gdf1*	-0.92	2.05
299	*Rragb*	-0.92	6.48

Rank	Gene	Log2(FC)	-LOG10(p-value)
300	*Gpr17*	-0.92	1.59
301	*Shank2*	-0.92	4.09
302	*Chadl*	-0.91	2.62
303	*Sept8*	-0.91	4.10
304	*Tchh*	-0.90	2.14
305	*Parvb*	-0.89	3.06
306	*Tdpoz4*	-0.89	4.50
307	*Ucn2*	-0.89	2.04
308	*Pabpc1l*	-0.88	1.31
309	*Lrrn2*	-0.88	1.54
310	*Tbx21*	-0.88	1.52
311	*Crybg3*	-0.88	3.03
312	*Fam102b*	-0.88	2.65
313	*Mfap3l*	-0.87	1.47
314	*Pcdhb16*	-0.87	2.36
315	*Thpo*	-0.87	2.12
316	*Nipsnap1*	-0.86	1.62
317	*Tmem169*	-0.86	1.66
318	*I730030J21Rik*	-0.86	3.20
319	*Fhl1*	-0.85	2.25
320	*Clvs1*	-0.85	1.77
321	*4933417G07Rik*	-0.85	2.74
322	*Gmip*	-0.84	3.96
323	*Kcnab3*	-0.84	1.64
324	*Gm25500*	-0.84	2.40

Rank	Gene	Log2(FC)	-LOG10(p-value)
325	*Secisbp2l*	-0.84	2.70
326	*Fli1*	-0.83	1.92
327	*Mtr*	-0.83	3.11
328	*Fxyd7*	-0.82	1.59
329	*Tigd5*	-0.82	1.50
330	*Smarca1*	-0.82	1.62
331	*Eogt*	-0.82	3.23
332	*Pcdh1*	-0.82	3.79
333	*Cplx2*	-0.82	1.51
334	*Adam19*	-0.82	2.78
335	*Rundc3a*	-0.81	2.36
336	*Egr1*	-0.81	1.36
337	*Gm29682*	-0.81	2.57
338	*Rnf144a*	-0.80	3.63
339	*Plagl1*	-0.80	2.76
340	*Nxpe4*	-0.80	1.89
341	*Fbxl22*	-0.80	3.31
342	*Plk3*	-0.79	1.92
343	*Gcnt2*	-0.78	1.97
344	*4921513I03Rik*	-0.77	1.64
345	*Tenm3*	-0.77	2.10
346	*Tspyl2*	-0.77	3.76
347	*Calcrl*	-0.77	1.67
348	*Rmdn2*	-0.77	1.96
349	*Sept3*	-0.77	2.16

Rank	Gene	Log2(FC)	-LOG10(p-value)
350	*Erg*	-0.77	1.91
351	*Adora2a*	-0.76	2.06
352	*Ndufa4l2*	-0.76	2.25
353	*Sec31b*	-0.76	1.34
354	*Slc25a47*	-0.75	1.39
355	*Slc6a17*	-0.75	2.02
356	*Abcb6*	-0.75	2.93
357	*Stx1b*	-0.75	1.97
358	*1810011H11Rik*	-0.75	1.71
359	*Dancr*	-0.75	2.47
360	*Cacna1a*	-0.74	1.52
361	*Sparcl1*	-0.74	1.94
362	*Ankrd45*	-0.74	1.37
363	*Pawr*	-0.74	2.51
364	*Cep170*	-0.73	1.60
365	*Socs3*	-0.72	1.42
366	*Gata6*	-0.72	1.46
367	*Osbp2*	-0.72	1.39
368	*Plekhb1*	-0.72	1.40
369	*S100pbp*	-0.72	3.08
370	*Degs2*	-0.71	2.39
371	*Card14*	-0.70	2.22
372	*Serping1*	-0.69	1.34
373	*Cd34*	-0.69	2.33
374	*Slc43a3*	-0.69	2.79

Rank	Gene	Log2(FC)	-LOG10(p-value)
375	*Nphp4*	-0.69	1.92
376	*Hyal1*	-0.69	1.86
377	*Tmem86b*	-0.69	1.88
378	*Adssl1*	-0.68	3.28
379	*Ppm1f*	-0.68	2.94
380	*Nhs*	-0.67	1.79
381	*Pcdhb22*	-0.67	2.14
382	*Zbtb46*	-0.67	2.33
383	*Snhg8*	-0.67	1.34
384	*Nacad*	-0.67	1.73
385	*Ugp2*	-0.66	2.08
386	*Prss53*	-0.66	1.47
387	*Myocd*	-0.66	1.88
388	*Myct1*	-0.65	1.67
389	*Zc4h2*	-0.65	2.24
390	*Mpp1*	-0.65	2.64
391	*Tert*	-0.64	2.34
392	*Gimap4*	-0.64	1.61
393	*Casp12*	-0.63	1.59
394	*Spire1*	-0.63	2.19
395	*Glce*	-0.63	2.31
396	*Six5*	-0.63	2.85
397	*Kif5a*	-0.62	1.87
398	*Scarf1*	-0.62	3.62
399	*Lime1*	-0.62	2.07

Rank	Gene	Log2(FC)	-LOG10(p-value)
400	*Tmem181b-ps*	-0.62	1.81
401	*Gm16576*	-0.62	1.92
402	*Zfp366*	-0.62	2.26
403	*Tshz2*	-0.61	1.77
404	*Kcnq4*	-0.61	1.76
405	*4933439C10Rik*	-0.61	1.87
406	*Mical1*	-0.61	1.93
407	*Nmnat1*	-0.61	1.38
408	*Ccnjl*	-0.61	1.62
409	*Fam180a*	-0.61	1.70
410	*Ckb*	-0.60	1.67
411	*Zfp108*	-0.60	1.98
412	*Nacc2*	-0.60	2.26
413	*Tmem165*	-0.60	3.24
414	*Gm9079*	-0.60	1.31
415	*Dzip1*	-0.59	2.42
416	*Acsl6*	-0.59	1.70
417	*Psip1*	-0.59	1.89
418	*Mgat3*	-0.59	2.26
419	*Bach2*	-0.59	1.34
420	*Gramd1c*	-0.59	1.74
421	*2410006H16Rik*	-0.59	2.16
422	*Mum1l1*	-0.59	1.51
423	*Mllt6*	-0.59	3.05
