# Supplementary material for: CFP1 governs uterine epigenetic landscapes to intervene in progesterone responses for uterine physiology and suppression of endometriosis
Source: Nat Commun. 2023 Jun 3;14:3220. doi: 10.1038/s41467-023-39008-0 (PMC10239508; doi:10.1038/s41467-023-39008-0)
Supplement: Supplementary file 5 — Reporting Summary [file 41467_2023_39008_MOESM5_ESM.pdf]

nature portfolio

Corresponding author(s): Haengseok Song

Last updated by author(s): Apr 15, 2023

Reporting Summary

Nature Portfolio wishes to improve the reproducibility of the work that we publish. This form provides structure for consistency and transparency in reporting. For further information on Nature Portfolio policies, see our Editorial Policies and the Editorial Policy Checklist.

Statistics

For all statistical analyses, confirm that the following items are present in the figure legend, table legend, main text, or Methods section.

n/a	Confirmed	
☐	☒	The exact sample size (*n*) for each experimental group/condition, given as a discrete number and unit of measurement
☐	☒	A statement on whether measurements were taken from distinct samples or whether the same sample was measured repeatedly
☐	☒	The statistical test(s) used AND whether they are one- or two-sided<br>*Only common tests should be described solely by name; describe more complex techniques in the Methods section.*
☐	☒	A description of all covariates tested
☐	☒	A description of any assumptions or corrections, such as tests of normality and adjustment for multiple comparisons
☐	☒	A full description of the statistical parameters including central tendency (e.g. means) or other basic estimates (e.g. regression coefficient) AND variation (e.g. standard deviation) or associated estimates of uncertainty (e.g. confidence intervals)
☒	☐	For null hypothesis testing, the test statistic (e.g. *F*, *t*, *r*) with confidence intervals, effect sizes, degrees of freedom and *P* value noted<br>*Give P values as exact values whenever suitable.*
☒	☐	For Bayesian analysis, information on the choice of priors and Markov chain Monte Carlo settings
☒	☐	For hierarchical and complex designs, identification of the appropriate level for tests and full reporting of outcomes
☐	☒	Estimates of effect sizes (e.g. Cohen's *d*, Pearson's *r*), indicating how they were calculated

Our web collection on statistics for biologists contains articles on many of the points above.

Software and code

Policy information about availability of computer code

Data collection	No software was used.
Data analysis	GSEA (20180602), Image Lab (6.0), GraphPad Prism 8, IGV (2.14.0), MeV (4 9 0), R program, Bowtie 2 (ver 2.3.4.2, PMID: 22388286), MACS2 (ver 2.1.1, PMID: 18798982), Deeptools (ver 3.4.1, PMID: 27079975), UCSC table browser (PMID: 14681465), Homer (ver 4.11.1, PMID: 20513432)

For manuscripts utilizing custom algorithms or software that are central to the research but not yet described in published literature, software must be made available to editors and reviewers. We strongly encourage code deposition in a community repository (e.g. GitHub). See the Nature Portfolio guidelines for submitting code & software for further information.

Data

Policy information about availability of data

All manuscripts must include a data availability statement. This statement should provide the following information, where applicable:

- Accession codes, unique identifiers, or web links for publicly available datasets
- A description of any restrictions on data availability
- For clinical datasets or third party data, please ensure that the statement adheres to our policy

Raw data files are deposited in the NCBI Gene Expression Omnibus database under Super Series accession number GSE219104. GSE219104 (https://www.ncbi.nlm.nih.gov/projects/geo/query/acc.cgi?acc=GSE219104). Public on May 10, 2023. Source data are provided with this paper.

Research involving human participants, their data, or biological material

Policy information about studies with human participants or human data. See also policy information about sex, gender (identity/presentation), and sexual orientation and race, ethnicity and racism.

Reporting on sex and gender	We studied the uterine tissue that only exists in women. Therefore, we received tissue from women.
Reporting on race, ethnicity, or other socially relevant groupings	The participants were recruited from women suffering from endometriosis aged 25 to 35. There is no outline of any self-selection bias.
Population characteristics	Endometriotic lesions were obtained from women (aged 25 to 35 years) suffering from endometriosis, confirmed by laparoscropy and histopathology. Patients had regular menstrual cycles and recruited without hormone treatment for at least 3 months before surgery.
Recruitment	Patient underwent hysteroscopy-laparoscopy surgery, and all participants signed the informed consent.
Ethics oversight	The sample application was approved by the ethics committee (XMYY-2021KYSB044)

Note that full information on the approval of the study protocol must also be provided in the manuscript.

Field-specific reporting

Please select the one below that is the best fit for your research. If you are not sure, read the appropriate sections before making your selection.

☒ Life sciences ☐ Behavioural & social sciences ☐ Ecological, evolutionary & environmental sciences

For a reference copy of the document with all sections, see nature.com/documents/nr-reporting-summary-flat.pdf

Life sciences study design

All studies must disclose on these points even when the disclosure is negative.

Sample size	All analyses were calculated with at least three to eight samples per group.
Data exclusions	No data were excluded from the analyses.
Replication	All experiments are reproducible. All experiments were organized through at least three repetitions. A detailed description of the replicate experiment was written in each figure legend.
Randomization	All samples were allocated randomly.
Blinding	The investigators were blinded to group allocation during data collection and analysis.

Reporting for specific materials, systems and methods

We require information from authors about some types of materials, experimental systems and methods used in many studies. Here, indicate whether each material, system or method listed is relevant to your study. If you are not sure if a list item applies to your research, read the appropriate section before selecting a response.

Materials & experimental systems

n/a	Involved in the study	
☐	☒	Antibodies
☒	☐	Eukaryotic cell lines
☒	☐	Palaeontology and archaeology
☐	☒	Animals and other organisms
☒	☐	Clinical data
☒	☐	Dual use research of concern
☒	☐	Plants

Methods

n/a	Involved in the study	
☐	☒	ChIP-seq
☒	☐	Flow cytometry
☒	☐	MRI-based neuroimaging

Antibodies

Antibodies used	We mentioned the dilution used for each antibody in Supplementary Table 5. CGBP, 1:200 - 1:1500, Abcam, ab198977; Acetylated tubulin, 1:500, Sigma, T7451; PGR, 1:200 - 1:1000, Thermo, MA1-410; ESR, 200 - 1:1000, Santa Cruz, sc-542; KI67, 1:200, Abcam,

	ab16667; GAPDH, 1:2000, Cell Signaling, 2118; CGBP, 5mg, Abcam, ab56035; H3K4me3, 5mg, Active motif, 39159; SET1, 5mg, Abcam, ab70378; Normal rabbit IgG, 5mg, Santa Cruz, sc-2027.<br><br>We mentioned the dilution used for each antibody in Supplementary Table 6. anti-rabbit IgG(H+L) conjugated HRP, 1:200 - 1:3000, Invitrogen, #3120, IHC/WB; anti-mouse IgG(H+L) conjugated HRP, 1:200 - 1:3000, Thermo, NC11430KR, IHC/WB; anti-rabbit IgG(H+L) conjugated Alexa Fluor 488, 1:200, Invitrogen, A-11008, IF; anti-mouse IgG(H+L) conjugated Alexa Fluor 488, 1:200, invitrogen, A-11001, IF; anti-rabbit IgG(H+L) conjugated Alexa Fluor 594, 1:200, invitrogen, A-11012, IF.
Validation	Antibody; Host; Reactivity; Applications<br>CGBP; Rabbit; mouse, rat, human; IHC,IF,WB<br>Acetylated tubulin; Mouse; mouse, human, rat, zebrafish; IF<br>PGR; Mouse; bovine, human, mouse, non-human primate, rat; IF, WB<br>ESR; Rabbit; ;IF,WB<br>KI67; Rabbit; mouse, human; IF<br>GAPDH; Rabbit; human, mouse, rabbit, monkey, bovine, pig; WB<br>CGBP; Rabbit; bovine, domestic dog, goat, guinea pig, zebrafish, horse, domesticated cat, human, house mouse, rabbit, rat; ChIP<br>H3K4me3, Rabbit; budding yeast, human, mouse; ChIP<br>SET1, Rabbit; mouse, rat, human; ChIP<br>Normal rabbit IgG; rabbit; ChIP

Animals and other research organisms

Policy information about studies involving animals; ARRIVE guidelines recommended for reporting animal research, and Sex and Gender in Research

Laboratory animals	Adult (8-10weeks of age) C57BL/6, Cfp1 f/f, Cfp1 d/d mice were housed in CHA University. All mice used in this study were housed under temperature- and light-controlled conditions with the light on for 12 hours daily and fed ad libitum.
Wild animals	Wild animals were not used in this study.
Reporting on sex	Cfp1 f/f mice were mated to PR cre/+ mice to generate Cfp1 f/+;PR cre/+ mice. Then, these mice were crossed to generate Cfp1 f/f and Cfp1 d/d mice. The male mice were used for mating, and female mice were used for the main results.
Field-collected samples	No field-collected samples were used in the study.
Ethics oversight	All mice used in this study were maintained in accordance with the policies of the CHA University Institutional Animal Care and Use Committee (IACUC 150083).

Note that full information on the approval of the study protocol must also be provided in the manuscript.

Plants

Seed stocks	*Report on the source of all seed stocks or other plant material used. If applicable, state the seed stock centre and catalogue number. If plant specimens were collected from the field, describe the collection location, date and sampling procedures.*
Novel plant genotypes	*Describe the methods by which all novel plant genotypes were produced. This includes those generated by transgenic approaches, gene editing, chemical/radiation-based mutagenesis and hybridization. For transgenic lines, describe the transformation method, the number of independent lines analyzed and the generation upon which experiments were performed. For gene-edited lines, describe the editor used, the endogenous sequence targeted for editing, the targeting guide RNA sequence (if applicable) and how the editor was applied.*
Authentication	*Describe any authentication procedures for each seed stock used or novel genotype generated. Describe any experiments used to assess the effect of a mutation and, where applicable, how potential secondary effects (e.g. second site T-DNA insertions, mosiacism, off-target gene editing) were examined.*

ChIP-seq

Data deposition

☒ Confirm that both raw and final processed data have been deposited in a public database such as GEO.

☒ Confirm that you have deposited or provided access to graph files (e.g. BED files) for the called peaks.

Data access links<br>*May remain private before publication.*	GSE219104 (https://www.ncbi.nlm.nih.gov/projects/geo/query/acc.cgi?acc=GSE219104).<br>Secure token : epcdwequbtchdkp
Files in database submission	CFP1 ChIP seq BL6-WT-PD4-IP.fastq.gz<br>CFP1 ChIP seq BL6-WT-PD4-Input.fastq.gz<br>H3k4me3 ChIP seq Cfp1 WT-IP.fastq.gz<br>H3k4me3 ChIP seq Cfp1 wt-input.fastq.gz<br>H3k4me3 ChIP seq Cfp1 cKO-IP.fastq.gz<br>H3k4me3 ChIP seq Cfp1 cKO-input.fastq.gz

CFP1 ChIP seq BL6-WT-PD4-IP_peaks.txt
CFP1 ChIP seq BL6-WT-PD4-input_peaks.txt
H3k4me3 ChIP seq Cfp1 WT IP_peaks.txt
H3k4me3 ChIP seq Cfp1 WT input_peaks.txt
H3k4me3 ChIP seq Cfp1 cKO IP_peaks.txt
H3k4me3 ChIP seq Cfp1 cKO input_peaks.txt

Genome browser session
(e.g. UCSC)

No longer applicable

Methodology

Replicates

Each ChIP-seq experiment was performed on the pools of uterine cells excluding smooth muscle cells collected from the uterus of 3 to 6 mice.

Sequencing depth

Single-end 75bp

Antibodies

CGBP, Abcam, ab56035, ChIP; H3K4me3, Active motif, 39159, ChIP; SET1, Abcam, ab70378, ChIP; Normal rabbit IgG, Santa Cruz, sc-2027, ChIP

Peak calling parameters

Macs2 Call peak - no control, narrow peak, q value 0.05

Data quality

CFP1 ChIP seq BL6-WT-PD4-IP : effective genome size = 2.70e+09, band width = 300, qvalue cutoff = 5.00e-02, Broad region calling is off, tag size is determined as 33 bps, total tags in treatment: 16739379
CFP1 ChIP seq BL6-WT-PD4-Input : effective genome size = 2.70e+09, band width = 300, qvalue cutoff = 5.00e-02, Broad region calling is off, tag size is determined as 33 bps, total tags in treatment: 17119393
H3k4me3 ChIP seq Cfp1 WT-IP : effective genome size = 1.87e+09, band width = 300, qvalue cutoff = 5.00e-02, Broad region calling is off, tag size is determined as 49 bps, total tags in treatment: 45914279
H3k4me3 ChIP seq Cfp1 wt-input : effective genome size = 1.87e+09, band width = 300, qvalue cutoff = 5.00e-02, Broad region calling is off, tag size is determined as 47 bps, total tags in treatment: 25358286
H3k4me3 ChIP seq Cfp1 cKO-IP : effective genome size = 1.87e+09, band width = 300, qvalue cutoff = 5.00e-02, Broad region calling is off, tag size is determined as 47 bps, total tags in treatment: 43841920
H3k4me3 ChIP seq Cfp1 cKO-input : effective genome size = 1.87e+09, band width = 300, qvalue cutoff = 5.00e-02, Broad region calling is off, tag size is determined as 46 bps, total tags in treatment: 36827683

Software

bowtie2 (ver 2.3.4.2, PMID: 22388286), deeptools (ver 3.4.1, PMID: 27079975), UCSC table browser (PMID:14681465)Using seqMINER (ver 1.3.4; PMID: 21177645), CFP1 ChIP-seq signal distribution was observed around mm10 TSSs. From the result of k-means clustering into three clusters, the cluster with the most CFP1 ChIP-seq signal was selected and 143bp (average ChIP-seq peak length; MACS2 ver 2.1.1, default setting; PMID: 18798982) centered on the selected TSSs were put through motif analysis using HOMER (ver 4.11.1, PMID: 20513432).
